# Models of social prescribing to address non-medical needs in adults: a scoping review

**DOI:** 10.1186/s12913-023-09650-x

**Published:** 2023-06-15

**Authors:** Candice Oster, Claire Skelton, Richard Leibbrandt, Sonia Hines, Billie Bonevski

**Affiliations:** 1 College of Nursing & Health Sciences, Caring Futures Institute, Flinders University, GPO Box 2100, Adelaide, SA 5001 Australia; 2 College of Medicine & Public Health, Flinders University, Adelaide, SA Australia; 3 College of Science & Engineering, Flinders University, Adelaide, SA Australia; 4 College of Medicine & Public Health, Flinders Rural and Remote Health, Flinders University, Alice Springs, Northern Territory Australia

**Keywords:** Social prescribing, Community referral, Social determinants of health, Scoping review

## Abstract

**Background:**

The health and wellbeing consequences of social determinants of health and health behaviours are well established. This has led to a growing interest in social prescribing, which involves linking people to services and supports in the community and voluntary sectors to address non-medical needs. However, there is considerable variability in approaches to social prescribing with little guidance on how social prescribing could be developed to reflect local health systems and needs. The purpose of this scoping review was to describe the types of social prescribing models used to address non-medical needs to inform co-design and decision-making for social prescribing program developers.

**Methods:**

We searched Ovid MEDLINE(R), CINAHL, Web of Science, Scopus, National Institute for Health Research Clinical Research Network, Cochrane Central Register of Controlled Trials, WHO International Clinical Trial Registry Platform, and ProQuest – Dissertations and Theses for articles and grey literature describing social prescribing programs. Reference lists of literature reviews were also searched. The searches were conducted on 2 August 2021 and yielded 5383 results following removal of duplicates.

**Results:**

148 documents describing 159 social prescribing programs were included in the review. We describe the contexts in which the programs were delivered, the program target groups and services/supports to which participants were referred, the staff involved in the programs, program funding, and the use of digital systems.

**Conclusions:**

There is significant variability in social prescribing approaches internationally. Social prescribing programs can be summarised as including six planning stages and six program processes. We provide guidance for decision-makers regarding what to consider when designing social prescribing programs.

**Supplementary Information:**

The online version contains supplementary material available at 10.1186/s12913-023-09650-x.

## Background

People’s health and wellbeing are affected by both medical and non-medical factors, with non-medical factors reported to account for over 80% of health outcomes [1]. Non-medical factors include social determinants of health, defined as, “the circumstances in which people are born, grow, live, work and age” ([2], p. 1), and health behaviours (such as alcohol and drug use, sexual activity, diet, and exercise) [1, 2]. Non-medical factors furthermore place additional burden on health systems and health practitioners, particularly General Practitioners (GPs). For example, data from the UK and Australia suggest around 20% of visits to GPs are for non-medical needs [3, 4]. Addressing non-medical needs is therefore increasingly recognised as important to support health and wellbeing and reduce burden on health systems [5].

One way to address non-medical needs is to link individuals with health and social supports in their community, such as housing support, financial services, domestic violence support, social groups, etc. This is commonly termed ‘social prescribing’ [6]. There are various definitions of social prescribing. Here we refer to the broad concept of social prescribing as a systematic approach that “enables a range of stakeholders, often based in healthcare, to refer individuals to non-clinical interventions, such as social activities and social services, to empower individuals and improve their health and wellbeing” ([7], p. 1).

The United Kingdom (UK) has been at the forefront of implementing social prescribing programs, with the United States of America (USA) also developing and implementing strategies for routine screening for non-medical needs, within and outside of health settings, and referral to community services. There is also growing interest in other European countries as well as Asia, Australia, Canada, and New Zealand [4, 7]. In Australia, for example, the Federal Government’s National Preventive Health Strategy identified social prescribing as one of seven enablers for mobilising a prevention system, with the aim for it to be “embedded in the health system at a local level” by 2030 ([8], p. 35). However, there is little guidance on how social prescribing could be developed for health systems outside of the UK and USA [7, 9].

There have been several systematic reviews of the social prescribing evidence-base (e.g., [6, 10–15]). While noting methodological limitations relating to scale and study design, these reviews have identified promising results for social prescribing programs in terms of improved health and wellbeing, day-to-day functioning, social contacts, health-related behaviours, and healthcare demand. The reviews furthermore identify significant variation in approaches to social prescribing [10].

Social prescribing can be a simple intervention in the form of an information service providing details about community supports, or a more complex intervention that incorporates working with a link work (also termed a navigator or community connector) to identify non-medical needs and connect individuals to relevant services. Social prescribing programs are typically delivered in health contexts, such as GP clinics, but can also be delivered in the community or online. Individuals can access programs through referral from health professionals or through self-referral, often mediated by a link worker. Programs also vary in their target populations, the types of non-medical needs they address, and the services to which individuals are referred. For example, programs might target the broader population or focus on specific target groups such as those with long-term physical or mental health problems [16, 17]. Programs might address a broad array of non-medical needs [11] or focus on specifical needs such as referring participants to community-based arts programs [18] or physical activity [19]. Further areas of variability in approaches to social prescribing include funding models [20] and the role of digital systems [21]. What is missing from our knowledge of social prescribing is an understanding of the various approaches to, and components of, social prescribing and how these are brought together in social prescribing program models internationally.

There has been limited research on models of social prescribing to date. Kimberlee [22] identified three broad approaches to social prescribing based on a literature review and interviews/focus groups with social prescribing practitioners, services users, and GPs. These are light, medium, and holistic. In another study, based on a workshop hosted by a Primary Care Trust in the UK, Brandling et al. [23] identified six models: 1. Information service; 2. Information and telephone line service; 3. Primary care referral to a social prescribing service; 4. Practice based generic referral worker; 5. Practice based specialist referral worker; 6. Non-primary care based. However, these studies focus solely on social prescribing delivered in the UK, with Kimberlee [22] only looking at models delivered in primary care. More recently, Morse et al. [7] described global developments in social prescribing across 17 countries, informed by interviews with social prescribing experts and iterative discussion and feedback with a working group of social prescribing practitioners. They identified essential social prescribing inputs as service delivery (social prescribing activities/ processes); target population(s) and local landscape of available services; workforce; leadership and governance; financing; technology; and information, learning and accountability. While providing valuable insight into the international landscape of social prescribing, further research is needed to systematically explore models of social prescribing.

Seventeen published systematic reviews of the literature on social prescribing were identified [6, 10–14, 20, 24–33]. Rather than conduct a further systematic review of the evidence-base for social prescribing, we were interested in systematically scoping the various components of social prescribing and how these are brought together into social prescribing programs internationally. The purpose is to inform the co-design of social prescribing programs and decision-making for social prescribing program developers.

As Tierney et al. [4] identified in their realist review of social prescribing in primary care, key stakeholder consultation is vital ensure ‘buy-in’ to complex interventions such as social prescribing. Co-design is one way to consult with key stakeholders (such health professionals and practice staff, consumers, providers of non-medical services, and service planners) to ensure the program meets their needs. Thomas et al. [24], for example, conducted a systematic review of social prescribing interventions applying co-design and co-productive approaches. They identified eight studies, the outcomes of which suggest co-design and co-production “can lead to positive well-being outcomes among communities” (p.3896). A key element of co-design is gaining “an initial impression of the … task to be addressed”, where literature reviews are frequently used to develop an understanding of “existing service solutions related to the topic in question” ([34], p. 1603). This information is then presented to key stakeholders in co-design workshops for their feedback and decision-making. Understanding the components of social prescribing in existing programs would provide important information for co-design.

One scoping review of components of social prescribing link worker pathways in the UK was found [35]. The authors identified a variety of components, which they developed into a taxonomy of intervention components relating to the target population, initial referral from health professionals, consultation with link workers, prescribed services and activities, and outcome measurement. However, no scoping reviews were identified that systematically explore and describe models of social prescribing outside of the UK. To address this gap, we conducted a scoping review of the peer-reviewed and grey literature to describe the types of social prescribing models used internationally to address non-medical needs.

The review question was: What types of social prescribing models are used to connect adults aged 18 years and older to non-medical services? Secondary questions were: (a) What are the contexts in which social prescribing programs have been delivered? (b) What population groups have been targeted/included? (c) What types of services/supports are individuals referred to? (d) What staff are involved in social prescribing programs? (e) What funding mechanisms are used to support social prescribing programs? (f) What is the potential role of digital systems in social prescribing programs?

## Methods

The scoping review was conducted following the JBI methodology for scoping reviews [36] in accordance with an a priori protocol registered on Open Science Framework [37]. Deviations from the protocol are outlined below. The scoping review is reported according to the Reporting Items for Systematic Reviews and Meta-Analyses Extension for Scoping Reviews (PRISMA-ScR) [38].

### Eligibility criteria

Eligibility criteria were determined according to the key elements of population/participant, concepts, and context. We included studies and reports of social prescribing programs that included adult participants aged 18 and over. Studies addressing non-medical needs of those under age 18 were excluded due to the different needs of younger people and the likely role of parents or carers as mediators in programs designed for those under age 18.

In terms of the concept of social prescribing, we defined this in the broad sense of programs with the primary focus of linking people with services and supports outside of the health system to meet their non-medical needs [7]. Social prescribing had to be the primary focus of the study/document and we excluded documents where social prescribing was an adjunct to another intervention, or the focus was on the non-medical intervention to which individuals were referred (e.g., describing an arts program). We also excluded studies focusing on medical system navigation without also addressing non-medical needs. Social prescribing programs in health and non-health contexts were included.

Social prescribing programs are designed within specific communities and health and social care systems. The system in which we aim to apply the knowledge from this scoping review is Australia, highlighting the need for translatable information on social prescribing models. Given differences in health care systems worldwide, we excluded models implemented in low- and middle-income countries as defined by the Development Assistance Committee and represented in a list of all countries and territories eligible to receive official development assistance (available at www.oecd.org).

### Information sources

We included a wide variety of sources reporting on social prescribing, including peer-reviewed full text literature reporting qualitative, quantitative, and mixed methods studies. We also included non-research peer-reviewed literature describing models of social prescribing. We excluded literature reviews that met the inclusion criteria but used these for the purposes of searching their reference lists for studies relevant for inclusion in this scoping review. Grey literature was included, including research reports, Masters, and PhD theses (honours theses were excluded), and unpublished clinical trials where the research has not also been published in a peer-reviewed journal. We excluded opinion papers and research protocols. Conference abstracts were included if there was sufficient detail about the social prescribing model reported.

### Search strategy

An initial limited search of Ovid MEDLINE(R) and Scopus was undertaken to identify articles on the topic. The text words contained in the titles and abstracts of relevant articles, and the index terms used to describe the articles, were used to develop a full search strategy for Ovid MEDLINE(R) in collaboration with a research librarian (see Additional File 1). The search strategy was adapted for each included database and information source.

The databases included Ovid MEDLINE(R), CINAHL, Web of Science, and Scopus. Sources of grey literature included National Institute for Health Research Clinical Research Network, Cochrane Central Register of Controlled Trials, WHO International Clinical Trial Registry Platform, and ProQuest – Dissertations and Theses (note modification to the original protocol regarding grey literature below). Only studies in English are included; however, no language limiters were used in the searches. No date limits were applied. The searches were conducted on 2 August 2021.

### Study/Source of evidence selection

All identified citations were collated and uploaded into Endnote X9 (Clarivate Analytics, PA, USA) and duplicates removed. Sources were then uploaded into Covidence systematic review software (Veritas Health Innovation, Melbourne, Australia). Following a pilot test, titles and abstracts were screened by two independent reviewers (CO & CS) for assessment against the inclusion criteria. Potentially relevant sources were retrieved in full, and their citation details imported into Covidence. The full texts of selected citations were assessed in detail against the inclusion criteria by the same two independent reviewers. Reasons for exclusion of sources of evidence at full text that did not meet the inclusion criteria were recorded. Any disagreements between the reviewers arising at each stage of the selection process were resolved through discussion.

### Data extraction and synthesis

Data were extracted in Covidence using a data extraction tool developed by the reviewers based on the JBI Scoping Review Data Extraction Instrument template [36]. The data extracted included specific details about the participants, concept, context, study methods, and key findings relevant to the review questions. A draft data extraction form was developed and included in the protocol. The form was independently pilot trialled by two reviewers (CO & CS) on two included papers and results compared to ensure consistency between reviewers. The draft data extraction tool was modified and revised as necessary during the process of extracting data. Modifications included adding extraction of the terminology used for the concept (social prescribing or other terminology), how non-medical services were identified for the program (e.g., by a link worker or pre-determined by the program designers), and whether staff in the program received training.

Thirty (20%) included documents were randomly selected and independently screened by two reviewers (CO & CS; see modifications to the protocol below). The reviewers met twice (once following the first 20 documents being screened, and again following screening of the additional 10) to discuss and resolve any discrepancies. The remaining references were divided, and data extracted by one reviewer. The data extraction form is provided in Additional File 2*.* Results were synthesised in relation to the scoping review’s primary and secondary questions described above.

### Modifications from the protocol

Three modifications were made to the original protocol. First, due to the large number of included sources, it was not feasible to screen the reference lists of all included sources of evidence for additional studies. Instead, only the reference lists of literature reviews were screened. Given the number of literature reviews identified (*n* = 31) and the recency of their publication (between 2017 and 2021), this approach was considered appropriate for identifying sources that might not have been captured in our search strategies. Second, and again due to the large number of sources included, we did not search Google Advanced and Google Scholar for grey literature as intended in our protocol. Instead, we used the references of literature reviews that included grey literature for this purpose. The final modification related to data extraction. It was originally intended that all included documents would be independently screened by two reviewers, which proved unfeasible with the large number of included studies. Instead, a random selection of 20% of included documents were independently extracted as described above.

## Results

**Fig. 1:**
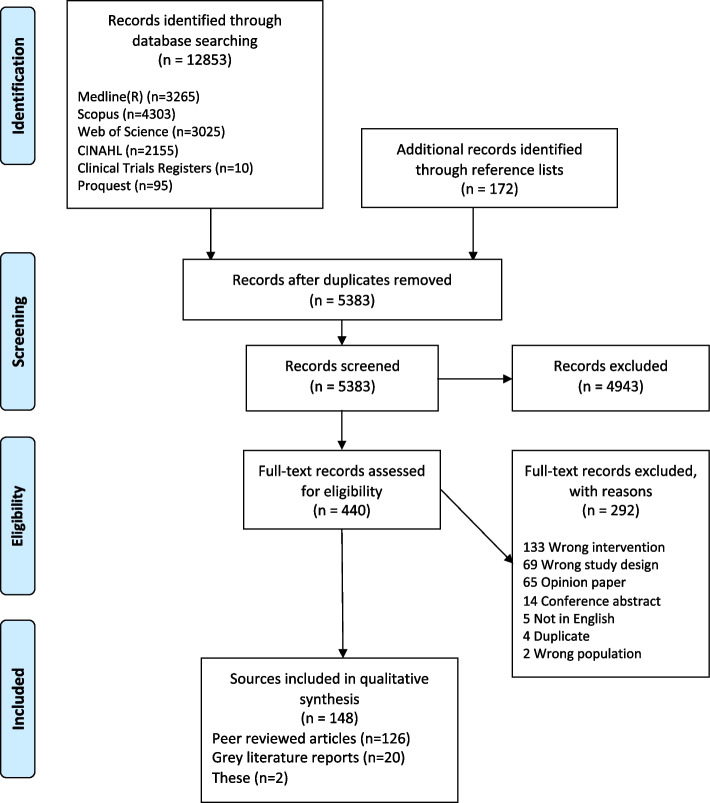
PRIMSA-SCR flowchart

A total of 5383 records were screened following removal of duplicates. A total of 4983 were excluded following title and abstract screening leaving 440 for full text screening. Following exclusion of 292 documents (with reasons, provided in Fig. 1), 148 were included in the scoping review and are summarised in Additional File 3. Four documents described more than one social prescribing program with a total of 159 social prescribing programs reported on.

**Fig. 2:**
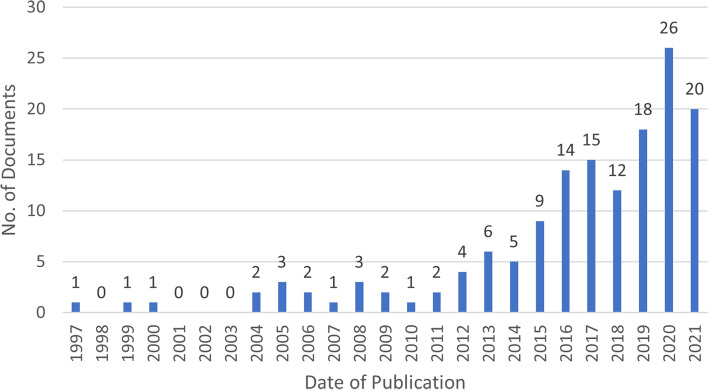
Number of documents by date of publication

Documents included in the scoping review were mainly peer reviewed journal articles (85.1%, *n* = 126) with 20 (13.5%) grey literature reports and two (1.4%) PhD theses. Date of publication of the included documents is summarised in Fig. 2, showing a growing interest in social prescribing from around 2013.

**Fig. 3:**
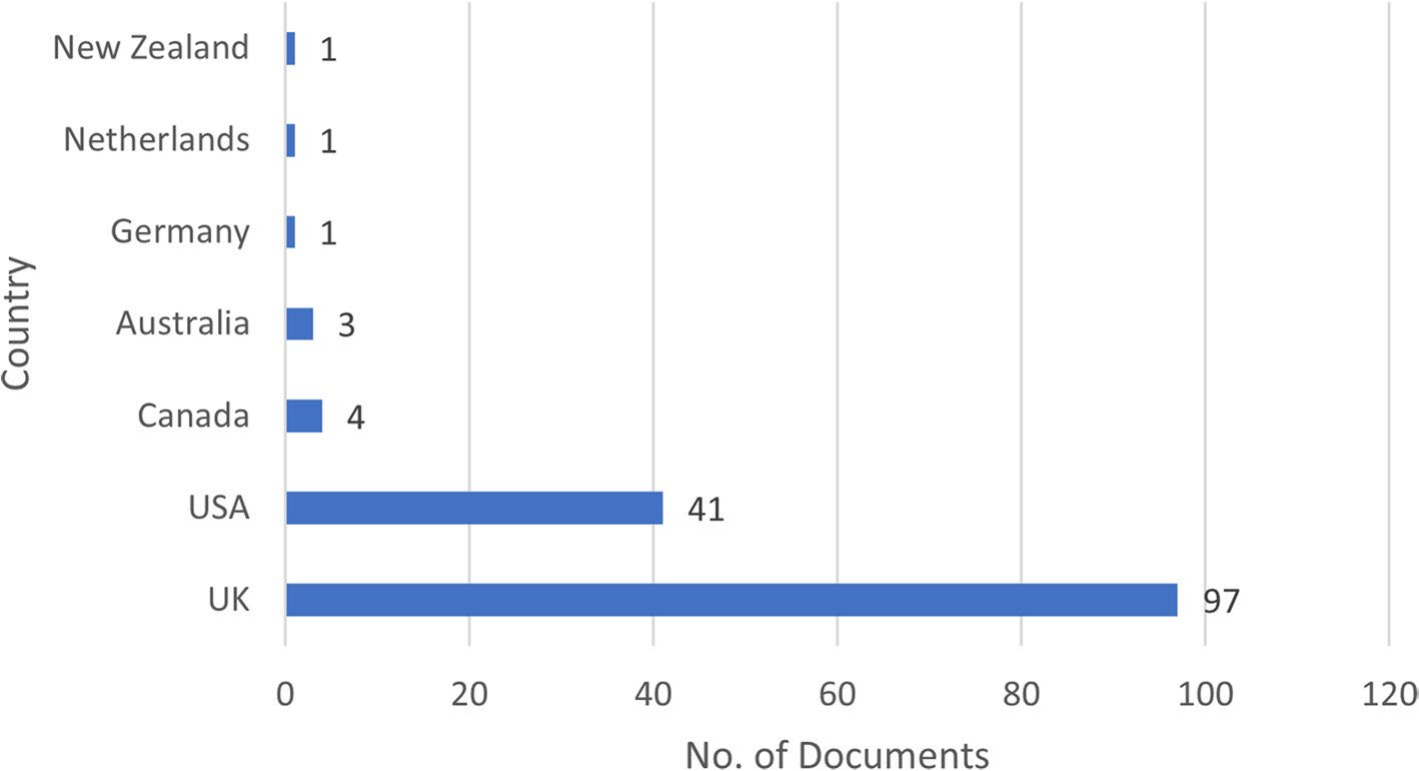
Country of origin of the documents

Country of origin is summarised in Fig. 3, showing most documents are from the UK (65.5%, *n* = 97) and USA (27.7%, *n* = 41). Methods used in documents reporting research were quantitative (33.8%, *n* = 50), mixed methods (31.1%, *n* = 46), and qualitative (25.0%, *n* = 37), with 10.1% (*n* = 15) describing or comparing social prescribing programs with no data collected.

**Fig. 4:**
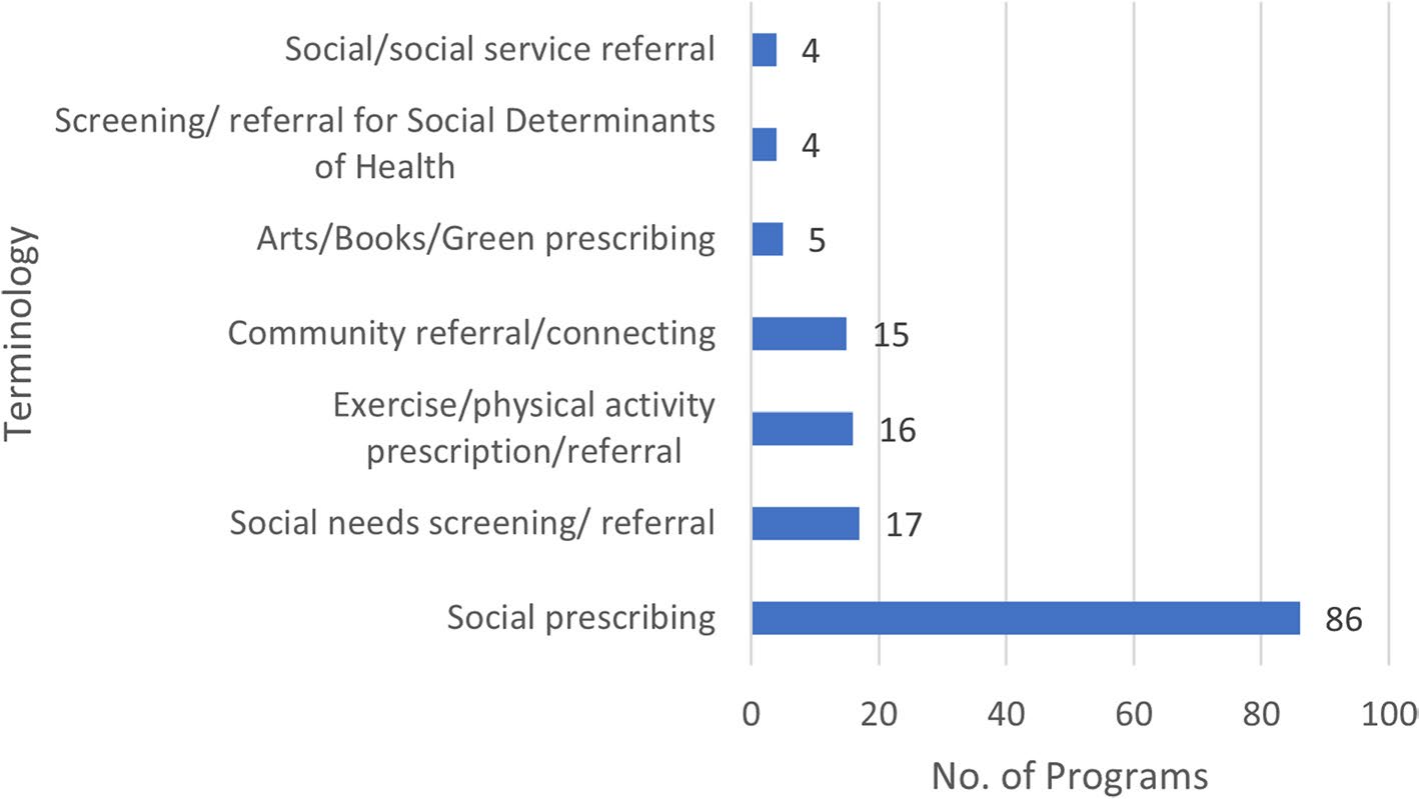
Commonly used terms used in the reported programs (Note: some programs use multiple terms)

The main terminology used in the included documents was social prescribing, with 58.1% of programs using this term (*n* = 86); other commonly used terms (used in more than two programs) are presented in Fig. 4.

Next, we summarise the included documents in relation to the secondary scoping review questions. We then discuss models of social prescribing to answer the primary question.

### What are the contexts in which social prescribing programs have been delivered?

**Fig. 5:**
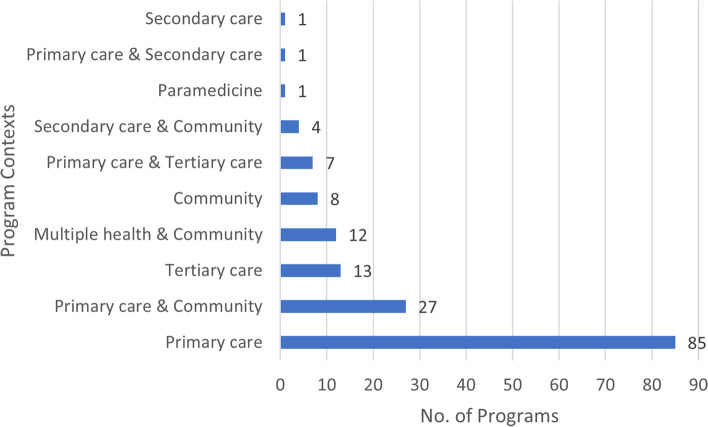
Contexts in which social prescribing programs were delivered

Social prescribing programs were primarily delivered through health contexts, mainly in primary care (78.0%, *n* = 124 included the primary care context in the program) (see Fig. 5). Documents also reported programs that were delivered in both health and community contexts (27.0%, *n* = 43), such as where the initial referral originated from a health context (primary, secondary, and/or tertiary care) with a link worker located in the community. Fewer (5.7%, *n* = 9) programs were located solely in the community (i.e., not linked to a health setting), with one provided by paramedics and four provided online.

### What population groups have been targeted/included?

**Fig. 6:**
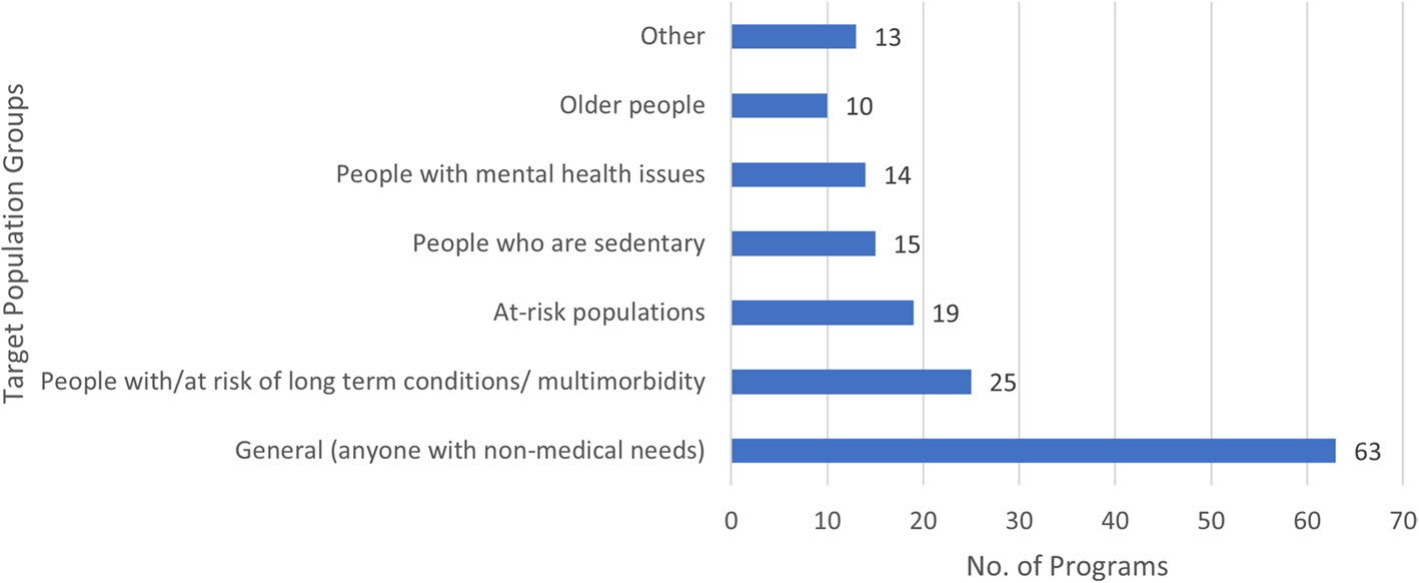
Population groups targeted/included in the reported programs

The data on populations groups was summarised into ‘general’, which refers to programs that supported anyone with non-medical needs in the catchment context, and programs targeting specific populations. Further, the targeted populations were categorised into the populations targeted in 10 or more programs (older people, people with mental health issues, people who are sedentary, people defined by the program as ‘at-risk’, people with or at-risk of long term conditions or multimorbidity) and an ‘other’ category, for those targeted in < 10 programs (specifically, parents/mothers (*n* = 3), people who have cancer (*n* = 3), people who experience loneliness (*n* = 2), young people (*n* = 2), carers (*n* = 1), people with unhealthy lifestyles (*n* = 1), and veterans (*n* = 1)). Some examples of population groups summarised in the general category of ‘at-risk’ include frequent attenders at health settings [39, 40], people with food insecurity [41], and vulnerable first-time young mothers [42]. As shown in Fig. 6, the category of ‘general’ was the most frequent category (39.6%, *n* = 63); however, it is important to note that there is likely to be overlap across the categories where, for example, people with long-term conditions might also have mental health problems or be older.

### What types of services/supports are individuals referred to?

**Fig. 7:**
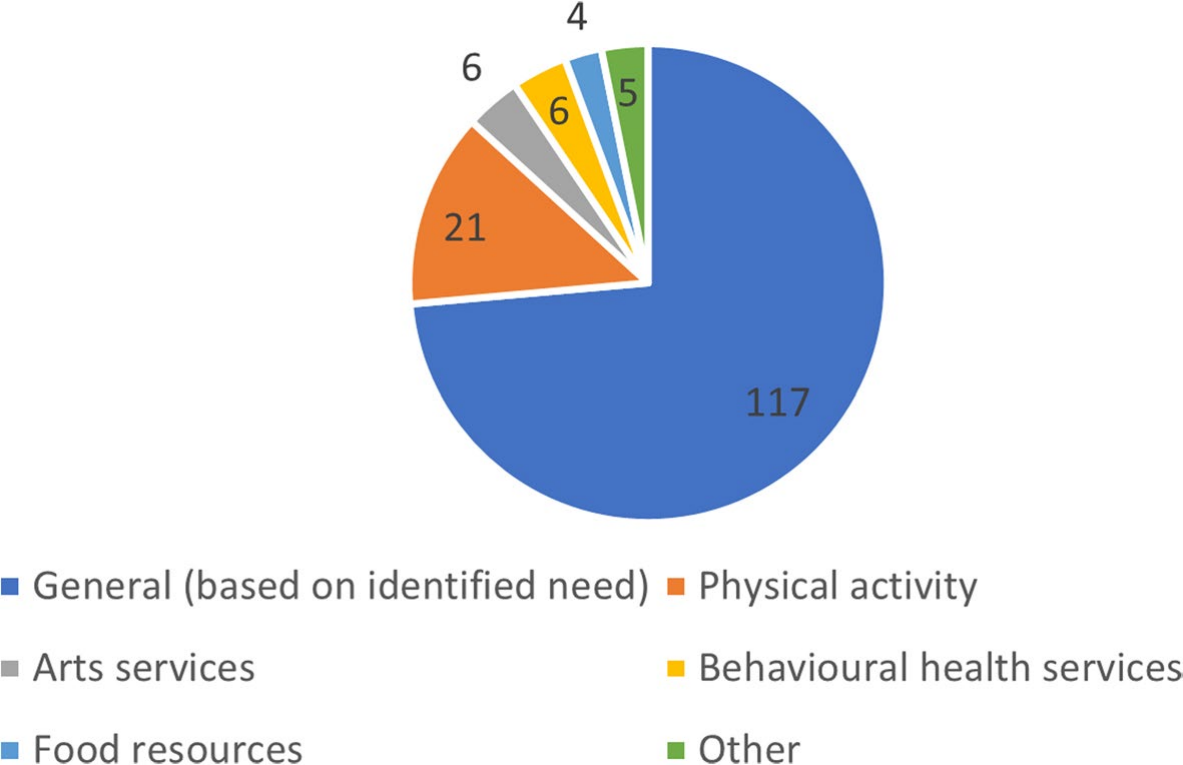
Types of services/supports included in the reported programs

Program participants were referred to a range of sources of support in the community and voluntary sectors. Programs either referred to a broad suite of services and supports (designated ‘general’ services/supports) or referred to services addressing specific non-medical needs, such as exercise and arts programs or referring only to food banks or homelessness services. Figure 7 shows the most commonly included services and supports, demonstrating that the majority (73.6%, *n* = 117) of programs referred to services and supports addressing non-medical needs in general. The category ‘other’ includes services addressing social isolation and homelessness, Cancer Council and wraparound services, and self-help books. Twenty-six (16.4%) programs used a screening tool to identify participants’ needs.

Identification of services/supports to which people could be referred was predominantly by either the program designers (referring to a specific service type or through service mapping of locally available non-medical services and supports; 57.2%, *n* = 91) or by those occupying a link worker role (using existing networks and/or building networks through their role; 40.3%, *n* = 64). Two programs used a combination of program designers and link workers, and no information was available on how services/supports were identified in a further two programs.

### What staff are involved in social prescribing programs?

Programs were staffed by healthcare service staff, social services staff, community-based organisation staff, staff hired to work in link worker/connector roles, volunteers, and students, either individually or in combination. People occupying link worker roles were either employed specifically for the program (e.g., someone with health coaching experience, an exercise professional, mental health professionals, social workers, or other health professionals) or used existing staff in community-based organisation (CBO) or health professionals employed in health settings. The most common staffing model was healthcare staff working in conjunction with a link worker(s) (73.0%, *n* = 116) followed by healthcare staff only (19.5%, *n* = 31). Four (2.5%) programs did not have any staff and used an online self-assessment and referral process instead, five (3.1%) programs only had a link worker, and staffing was unclear in three programs (1.9%). Fourteen (8.8%) programs included volunteers and two (1.3%) included students as link workers. A co-ordinator was employed in six (3.8%) programs.

The roles of staff in the programs included:

- Identifying potential program participants for non-medical needs screening
- Screening for non-medical needs (either using personal judgement or through a formal screening tool)
- Identifying non-medical services to support identified need
- Referral directly to non-medical services or to a link worker
- Providing individual support such as motivation and goal setting and/or facilitating service access and/follow-up (offered in 52.8% (*n* = 84) of programs).

Staff could undertake one or more of these roles depending on the specific program. For example, the program might involve health professionals identifying potential participants, screening, and referring directly to services and supports. Alternatively, the health professional might identify potential participants and refer them to a link worker. A link worker might screen for non-medical needs and refer to services with no follow-up or provide a more holistic service through additional support and follow-up. Programs might also offer the option for individuals to self-refer directly to link workers or to services.

Training was provided to staff in 45 programs (28.3%). This included training in health coaching (e.g., motivational interviewing and goal setting; 15 programs, 9.4%) or training in the social prescribing processes and tools used in the program (18.9%, *n* = 30). In 10 programs (6.3%), link workers were employed with existing skills or experience in psychotherapy, health coaching, and/or working in the health and social care sector.

### What funding mechanisms are used to support social prescribing programs?

Funding mechanisms were not mentioned in 45.3% (*n* = 72) of programs. Where funding was mentioned, Government funding was most frequently reported (41.1%, *n* = 65) followed by funding from charities (8.2%, *n* = 13). Other funding included combined government and charity funding (1.9%, *n* = 3), social impact bond (1.3%, *n* = 2), Community Based Organisation funding (1.3%, *n* = 2), and private company funds (1.3%, *n* = 2).

### What is the potential role of digital systems in social prescribing programs?

The use of digital technology was reported in 34.6% (*n* = 55) of programs. The most common use of digital technology was online non-medical needs screening and/or referral platforms (16.4%, *n* = 26), documenting social prescribing information in Electronic Medical Records (EMRs) (5.7%, *n* = 9), and the use of an online database of services (5.7%, *n* = 9).

### Models of social prescribing

**Fig. 8:**
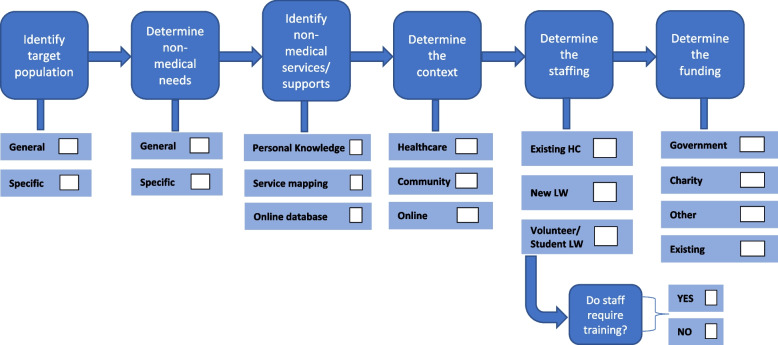
Social prescribing program planning

The data extracted from the included sources demonstrates the variability in approaches to social prescribing and the difficulty of articulating specific models for decision-makers to draw on. However, the social prescribing programs discussed above indicate six key aspects of social prescribing planning requiring decision-making for program designers, namely 1) which population(s) they will target, 2) which non-medical needs will be addressed, 3) how supports and services will be identified, 4) where the program will be delivered, 5) how the program will be staffed, and 6) how it will be funded. This is summarised in Fig. 8, with Table 1 presenting the decisions made against each stage of social prescribing planning in the programs included in this scoping review.

**Table 1:** Planning stages and decision-making options for each stage (according to programs included in the scoping review)

Planning stage	Options	Scoping review documents mapped against each option
1. Identify the target population for the program	General population: Broad reach to capture all those potentially affected by non-medical needs	[43–101]
	Specific population: E.g., relating to diagnosis, health behaviour (e.g., smoking, physical activity), or specific at-risk population with specific non-medical needs	[9, 39–42, 60, 68, 96, 102–185]
2. Determine the non-medical needs the program will address	General non-medical needs: Encompassing the wide range of social determinants and behavioural health	[9, 39, 40, 42–45, 48–50, 52, 53, 55–60, 62–89, 91, 92, 94–102, 106, 109, 111, 114, 116–121, 124–126, 130, 132, 134–136, 138–140, 142, 144–149, 153, 156, 158–163, 166, 167, 169–174, 176, 180–183, 185]
	Specific non-medical needs: E.g., sedentary lifestyle, homelessness, food insecurity, etc	[41, 46, 51, 54, 61, 90, 93, 96, 103–105, 107, 108, 110, 112, 113, 115, 122, 123, 127–129, 131, 133, 137, 141, 143, 146, 150–152, 154, 155, 157, 164, 165, 168, 175, 177–179, 184]
3. Identify appropriate non-medical services and supports	Personal knowledge (of Link Worker/ Other role)	[39, 40, 42, 45, 47, 48, 50, 52, 56–60, 62, 63, 65–74, 76, 80, 82, 84–87, 89, 91, 96, 98, 99, 112, 120, 124, 126, 130, 134, 135, 139, 140, 142, 144, 145, 149, 153, 156, 159, 161, 165, 172, 180–182]
	Service mapping	[9, 41, 43, 44, 46, 49, 51, 53–55, 61, 64, 68, 75, 77–79, 81, 83, 88, 90, 92–95, 97, 100–111, 113–119, 122, 123, 125, 127–129, 131–133, 136–138, 141, 143, 146–148, 150–152, 154, 155, 157, 158, 160, 162–164, 166–171, 173–179, 183–185]
	Use of an online service database	[49, 61, 68, 104, 159, 162, 166, 173, 185]
4. Determine the context in which the program will be delivered	Healthcare	[9, 39–41, 43–45, 47–59, 61–69, 73–78, 80, 82, 83, 89, 90, 92–97, 99, 101–106, 109, 110, 112, 114, 117–119, 122, 123, 127–129, 131–134, 136, 137, 141, 143–145, 147–151, 153–155, 159, 163, 168–170, 173–176, 179–184]
	Community	[18, 79, 100, 111, 130, 138–140, 171]
	Online	[79, 100, 111, 138]
	Mixed	[42, 46, 55, 60, 70, 71, 81, 84, 87, 88, 91, 98, 107, 108, 113, 115, 116, 120, 124–126, 135, 142, 146, 152, 156–158, 160, 164–167, 172, 177, 178, 185]
5. Determine how the program is to be staffed	Existing staff (health/social services/Community-Based Organisation)	[9, 39–74, 76, 78, 80–99, 101, 102, 104–108, 110, 112–137, 139–145, 147–158, 160–185]
	New link worker staff	[9, 39, 40, 42, 45, 47, 48, 50, 52–56, 58–60, 62, 63, 65–71, 73–75, 78, 80, 84–87, 89, 91, 97–99, 107, 110, 112, 114, 116–120, 122, 124–126, 130, 134–137, 139, 140, 142–145, 147–149, 151–157, 160, 161, 164, 165, 167, 170, 172, 176, 179–183]
	Volunteer/student link workers	[40, 48, 57, 77, 95, 101, 103, 109, 120, 130, 135, 139, 159, 169, 183]
5a. Do staff require training?	Yes	[9, 40–42, 45, 46, 53, 57–59, 66, 68, 69, 72, 76, 77, 83, 85, 87, 91, 95, 103–105, 110, 125, 126, 131, 133, 139, 147, 150, 152, 156, 157, 159, 163, 169, 171, 175, 176, 179, 181–183]
6. Determine how the program will be funded	Government funding	[9, 39, 40, 42, 45, 47, 50, 52–56, 58, 59, 63, 66, 68–70, 73, 74, 76, 80, 85–87, 89, 91, 97, 107, 108, 110, 113, 115–123, 125, 126, 135–137, 140, 143, 149, 153, 154, 157, 158, 160, 161, 163, 164, 170, 177, 178, 180–182]
	Charity	[84, 88, 113, 114, 130, 139, 145, 146, 152, 175, 177–179, 183]
	Other funding sources	[55, 65, 70, 98, 156, 166]
	Embed within existing services/funding	[41, 43, 44, 46, 48, 49, 51, 57, 61, 62, 64, 67, 71, 72, 75, 77, 81–83, 90, 92–96, 99–102, 104–106, 109, 111, 112, 124, 127–129, 131–134, 138, 141, 142, 144, 147, 148, 155, 159, 162, 165, 167–169, 171–174, 176, 184, 185]

**Fig. 9:**
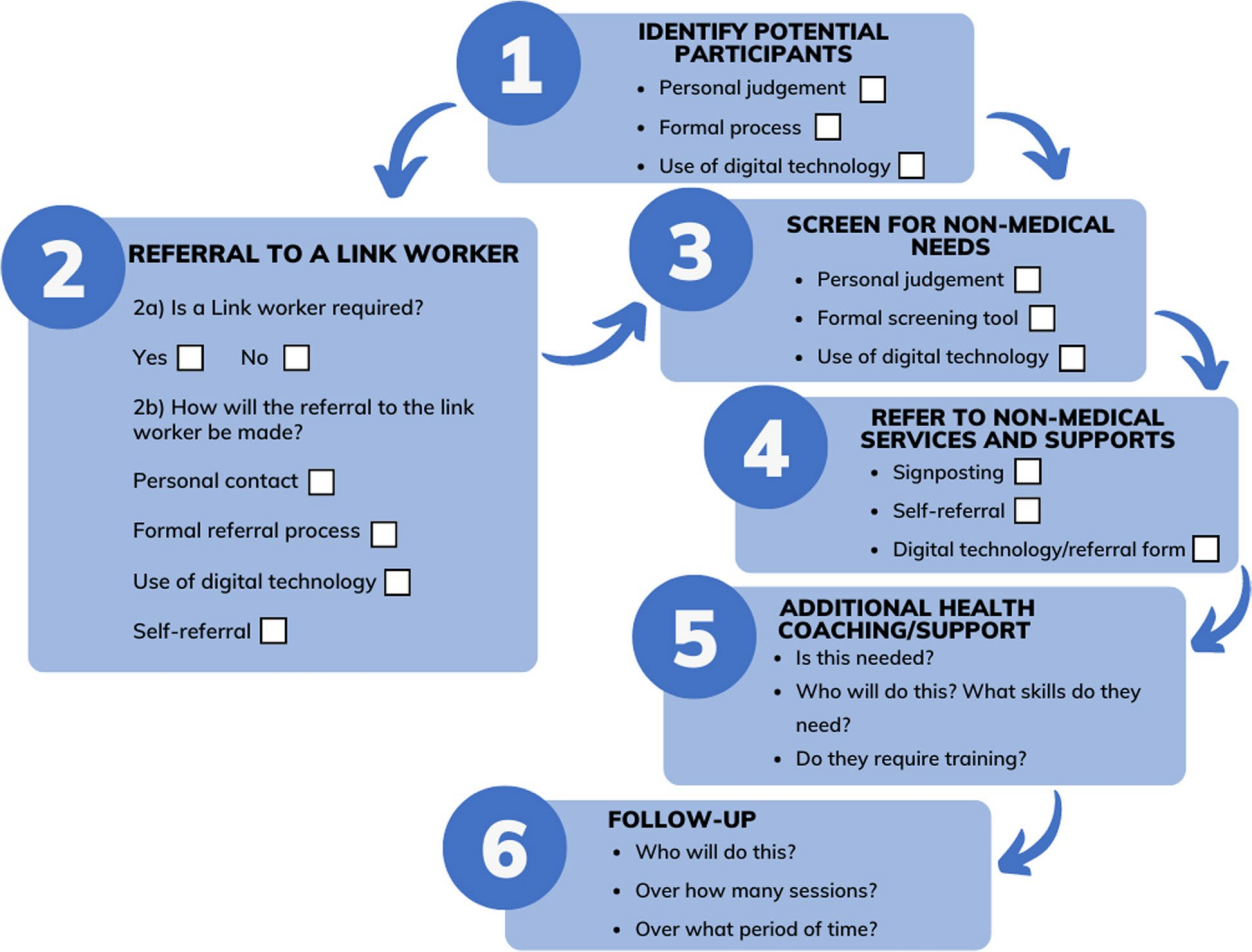
Social prescribing program processes

The programs analysed in the review furthermore suggest that that there are six areas related to the social prescribing process (or client journey) requiring decision-making for program designers, namely 1) how potential participants for the program will be identified, 2) whether and how they will be referred to a link worker, 3) how participants will be screened for non-medical needs, 4) how they will be referred to non-medical services and supports, 5) whether and how additional health coaching/support will be provided, and 6) whether and how follow-up will be provided. Programs can incorporate all or only some of these processes depending on local needs and systems. Figure 9 presents the social prescribing processes, with consideration for decision-makers provided in Table 2 based on the programs included in this scoping review.

**Table 2:** Program processes and decision-making options for each stage (according to programs included in the scoping review)

Process	Options	Scoping review documents mapped against each option
1. Identify potential participants	Personal/ professional judgement	[9, 39–42, 45–74, 76, 78–91, 94–101, 104–108, 111–114, 116–137, 139–158, 160–168, 172, 173, 175–183, 185]
	Formal process (e.g., screening tool)	[43, 44, 92, 93, 102, 105, 115, 159, 169–171, 184]
	Use of digital technology	[43, 44, 92, 93, 102, 110, 115, 169–171, 184]
2a. Is a Link Worker required	Yes	[9, 39–42, 46–48, 50, 52–60, 62, 63, 65–78, 80–82, 84–89, 91, 94–99, 101, 103, 106–108, 110, 112–122, 124–126, 130, 134–137, 139, 140, 142–145, 147–149, 151–157, 159–161, 164–167, 169–172, 175, 176, 179–183, 185]
2b. How will the referral to the link worker be made?	Personal contact	[9, 39, 40, 42, 47, 48, 50, 52–60, 62, 63, 66–74, 78, 80–82, 84–89, 91, 94–96, 98, 99, 101, 105–108, 110, 112–123, 125, 126, 130, 131, 134–136, 139, 140, 143–145, 148, 149, 151–157, 159–161, 164–167, 172, 175, 176, 179–183, 185]
	Formal referral process (e.g., referral form)	[41, 45, 46, 65, 68, 76, 97, 124, 137, 142, 147, 170, 171]
	Self-referral	[58, 60, 65, 68, 69, 78, 87, 91, 97–99, 124, 130, 135, 136, 139, 160, 167, 180]
	Use of digital technology	[75, 97, 142, 147, 169–171]
3. Screen for non-medical needs	Personal/ professional judgement	[9, 39, 40, 42, 45–47, 49–57, 60–63, 65–74, 76–78, 80–89, 97–99, 101, 104, 106–108, 110, 112, 113, 116–137, 139–161, 164–168, 171, 173, 177, 178, 181–185]
	Formal screening tool	[41, 43, 44, 48, 58, 59, 64, 75, 79, 82, 88, 92–94, 102, 103, 109, 111, 114, 115, 138, 162, 163, 169, 170, 172, 174–176]
	Use of digital technology	[41, 43, 44, 59, 75, 79, 82, 88, 92–94, 100, 102, 111, 115, 138, 163, 169, 170, 174–176]
4. Refer to non-medical services and supports	Provide service information to the individual (signposting)	[9, 39–44, 46–48, 50–53, 55–74, 76–78, 80–99, 101, 103–110, 112–137, 139–141, 143–149, 151–174, 176–185]
	Self-referral	[51, 111, 128, 129, 138, 158]
	Use of digital technology/ referral form	[43–45, 49, 54, 61, 75, 79, 82, 88, 94, 100, 102, 111, 138, 150, 160, 175]
5. Additional health coaching/ support	Yes	[9, 39, 40, 42, 45–47, 50, 55–58, 62, 66–74, 76, 78, 80, 84–89, 91, 96–99, 101, 103, 107, 114, 116, 120–122, 124, 126, 130, 134–136, 139, 140, 142–145, 147–149, 151–157, 160, 161, 164, 165, 172, 176, 181–183, 185]
6. Follow-up	Yes	[9, 39, 40, 42, 45–47, 50, 55, 56, 58, 62, 66–74, 76, 78, 80, 84–89, 91, 94–99, 101, 107, 109, 114, 116, 120–122, 124, 126, 130, 134, 135, 139, 140, 142–145, 147–149, 151–157, 160–162, 164–166, 172, 176, 179–183, 185]

Planning and processes are interlinked such that decision-making about what social prescribing processes (the model) will be used will affect decision-making about planning, such as staffing and funding.

## Discussion

This scoping review aimed to explore the broad types of social prescribing models that are used to connect adults aged 18 years and older to community and voluntary sector services for their non-medical needs. This is the first review that we are aware of to systematically examine international models of social prescribing. The review demonstrates growing interest in social prescribing and variability in social prescribing approaches and terminology.

In relation to the six secondary review questions (identifying social prescribing contexts, target populations, services/supports for referral, staffing, funding, and digital systems), the review demonstrates that a range of options are available to decision-makers when designing social prescribing programs. The included programs were predominantly delivered through health contexts, particularly primary care. This likely results from the high representation of programs in the UK where social prescribing through primary care is funded by the National Health Service (NHS) [186]. Social prescribing is also delivered through other health contexts, such as secondary and tertiary care, and less commonly is delivered across two health contexts (e.g., primary and secondary or primary and tertiary care). This highlights the potential for decision-makers to think beyond primary care when designing social prescribing programs to maximise access for people with non-medical needs. There is also a clear role for expanding social prescribing to include community and online contexts in addition to health settings, with nine programs delivered entirely in community contexts, including four which were online.

A further consideration is whether to target the broad population of people with non-medical needs or to focus on specific sub-populations, and also the criteria and processes for selecting suitable individuals. The included programs targeted a range of populations, from members of the population with non-medical needs in general to those with needs related specifically to a health condition or population group. Similar variability in social prescribing target populations was identified by Sandhu et al. [35]. Decisions around a target population for social prescribing might depend on the context in which the program is intended to be delivered (e.g., a mental health service), a needs analysis, the amount of funding available, or requirements of funding bodies to focus on specific populations such as older people or those with chronic physical or mental health conditions. Identification of non-medical needs in the target population can be through professional experience, screening tools, and/or self-identification by consumers.

Similarly, programs can involve referral to a wide range of non-medical supports, either targeting specific supports or, as was the case for approximately three quarters of the included programs, numerous supports addressing a range of needs. Again, contextual and funding factors, including what supports are available and accessible, and the population being targeted, would come into play for decision-makers around what services to prescribe. Decisions also need to be made about how to identify these supports (e.g., service mapping; existing networks and knowledge), where and how to house this information (e.g., online databases), and how the information is maintained (e.g., a funded position, use of volunteers, or knowledge and connections of link workers).

In terms of the staffing of social prescribing programs, the review demonstrates that while link workers play a key role in social prescribing (likely due to the dominance of UK-based programs in the review), there are additional staff that are included in social prescribing programs beyond the link worker role, such as health professionals and health service staff, students, volunteers, and co-ordinators. The importance of staff beyond the link worker role has also been identified by Sandhu et al. [35] in their scoping review of the link worker role. Other roles not discussed in this review but important to consider in social prescribing programs are peer workers and those who deliver the non-medical services to which people are prescribed. Husk et al. [186], for example, found that leaders of a social prescribing activity to which consumers are prescribed play an important role in maintaining adherence (ongoing attendance) to the activity.

Training is another important element of the staffing of social prescribing models. For example, in a systematic review of barriers and facilitators to implementing and delivering social prescribing services, Pescheny et al. [33] identified training of all social prescribing staff in the model and process as a facilitator, with lack of training being a barrier. Different staff will have different roles in a program, such as needs identification, referral, support, and follow-up, and training and supervision (or existing skills and qualifications) are needed around these roles (e.g., training in the social prescribing processes or health coaching). While not explored in our review, Husk et al. [186] furthermore noted the importance of the skills of leaders of social prescribing activities in developing and maintaining motivation. Provision of support to the staff involved in the program is also important, for example peer support by other link workers [187] or mental health training (such as mental health first aid) for those in link worker (or other) roles to manage mental health concerns (e.g., suicidality) that might arise.

Funding of the included programs was predominantly government funding. Funding was not mentioned in the reporting of nearly half of the reviewed programs. Given that 78% of programmes were delivered in health contexts in the UK (65%) and USA (28%), we might conclude that funding was primarily from health service sources. Sandhu et al. [188], for example, discuss the funding of social prescribing in the UK by the NHS and in the USA through Medicaid dollars and contracts with managed care organisations by the Centers for Medicare and Medicaid Services. However, it may not be the case that 100% of costs are provided in this way and there are many costs to consider when designing and implementing social prescribing programmes, including salaries and on-costs such as management, administration, and other back-office functions.

Finally, while digital systems were reported in just over a third of the included programs, decision-makers are alerted to the potential for use of online non-medical needs screening and/or referral platforms, documenting social prescribing information in Electronic Medical Records (EMRs), and the use of an online database of services. It is worth noting that absence of reporting on the secondary review questions in the included documents does not necessarily reflect the reality of program delivery. This is certainly the case with the role of EMRs in social prescribing in the UK (reported in 5.7% of programs) where the UK NHS has rolled out access to electronic medical records to link workers.

Looking at models of social prescribing across the programs included in the review (the primary research question), it is apparent that social prescribing programs are predominantly conducted in or through the primary care context, targeting members of the general population with non-medical needs, and referring them to a broad range of services and supports to address these needs. Staffing of social prescribing programs predominantly involves health care staff working in conjunction with a link worker to provide person-centred support, and the most common form of funding is through government funds. This is likely because the programs reported on are mostly from the UK, where this is the dominant model [10]. Griffiths et al. [12] recently conducted a systematic review of this primary care-link worker model of social prescribing. Eight studies were included in the review, all of which reported some positive outcomes for participants (e.g., quality of life, self-reported health status, well-being, patient activation, and relationships and social networks). Several weaknesses and limitations in study design were identified, including: “a lack of comparative controls, short duration and single point follow-up, a lack of standardised assessments, missing data, and a failure to consider potential confounding factors” (p.31).

Alternate models targeting the general population, often used in the USA, involve routinely screening patients across a range of health contexts for non-medical needs and the use of digital technology such as an online screening and referral platform to provide patients with a referral to non-medical services and supports [189]. Funding for these programs is generally incorporated into existing service funding. For example, Community Rx is a digital social prescribing model used in the USA [43, 44, 102]. It includes a database of community resources and an IT platform interfaced with electronic health records to generate a personalised list of community resources close to the person’s home. Community Rx can be adapted to various contexts and population groups and embedded into standard patient care. These two broad approaches to targeting the general population – intensive support through link workers versus a less intensive screening and referral process—are similar to the models described by Brandling et al. [23] and Kimberlee [22] and discussed earlier. They can be understood as two ends of a spectrum from what Kimberlee [22] calls social prescribing light and one end and social prescribing wholistic at the other.

Programs can also focus on targeted population groups (e.g., those with a particular diagnosis or health behaviour) and connect them with specific non-medical supports, such as food banks, housing support, or exercise programs. For example, Marpadga et al. [103] describe a hospital-based screening and referral program in the USA for food-insecure patients with diabetes. The program was embedded as a component of usual care and involved screening patients using the validated Hunger Vital Signs food insecurity screening tool. Those identified as food insecure were assessed by volunteers for eligibility to specific food resources and preferences regarding food, cooking, and transportation, and provided with tailored information about available community-based programs and resources.

By systematically scoping the broad range of social prescribing programs reported in the peer-reviewed and grey literature, our review provides greater depth of understanding of the elements that comprise social prescribing beyond the UK-based models discussed by Brandling et al. [23] and Kimberlee [22]. We summarise the varying approaches to social prescribing as comprising six planning stages and six social prescribing program processes. We also outline options for decision-makers across these stages and processes in terms of how existing social prescribing programs have been developed and implemented depending on their purpose and context. The planning and process elements identified in this scoping review are similar to the social prescribing inputs identified in Morse et al.’s [7] recent description of global developments in social prescribing, discussed earlier. Morse et al.’s work, like ours, demonstrates the importance of information on how social prescribing has been developed and implemented internationally to inform decision-making about social prescribing program development that reflects local contexts and systems.

There are also aspects of social prescribing that were not addressed in this review, but which are important from an implementation perspective. For example, the World Health Organisation [187] has provided guidance on how to implement link worker social prescribing that includes seven stages, namely 1) Conduct a situation analysis, 2) Assemble a core implementation team, 3) Develop an implementation workplan, 4) Map out community resources, 5) Get everyone on board, 6) Link worker training, and 7) Monitoring and evaluation. It is also important to consider the potential role of data collection through social prescribing to inform actions ‘up-stream’ in terms of needs-based asset building to ensure non-medical services and supports are available. Accessible and cost-effective non-medical services have been identified as important in ensuring consumer engagement in social prescribing [186].

Finally, our review highlights the need to consider different models of social prescribing when examining the social prescribing evidence-base. Existing reviews tend to focus on outcomes of a model of social prescribing implemented in a narrow context, such as focusing on primary care-link worker models in UK [10, 12]. For example, Husk et al. [186] conducted a realist review of four models of primary care-based social prescribing (signposting, direct referral from primary care, link worker, and wholistic), concluding “the evidence base is not sufficiently developed methodologically … to make any general inferences about effectiveness of particular models and approaches” (p. 309). It would also be worthwhile to compare outcomes of different models implemented in a single context or social prescribing models implemented across different contexts or population groups. Understanding the evidence-base regarding what works, how, for whom and in what context would provide useful information to social prescribing program designers.

### Limitations

Our scoping review is limited by the inclusion of only English language documents and lack of evidence on social prescribing models implemented in low- and middle-income countries. Although no documents from models implemented in low- and middle-income countries were identified, expanding the inclusion criteria to incorporate non-English language documents might have identified models in these countries. The search strategy was developed by a research librarian, and we aimed to encompass the broadest possible conceptualisation of social prescribing. However, given the variability of models/approaches to social prescribing, the wording used in our search strategy might not have captured all approaches to referring people to services and supports for non-medical needs and the use of additional terms might have resulted in additional programs being identified. The use of reference lists of literature reviews to identify grey literature is a further limitation.

## Conclusion

Non-medical needs relating to social determinants of health and health behaviours have a significant effect on health and wellbeing. Social prescribing programs aim to address non-medical needs by connecting people to services and supports in the community and voluntary sectors. Our scoping review has identified that social prescribing programs are frequently implemented in the UK and USA with a variety of approaches to social prescribing adopted. We have summarised the components of social prescribing described in the literature into six planning stages and six program processes. Those planning and implementing social prescribing programs should consider the applicability of the various components of social prescribing models identified in this review to their setting and purpose.

## Supplementary Information

**Additional file 1.** Search strategy.

**Additional file 2.** Data extraction instrument.

**Additional file 3.** Data summary table.

## Data Availability

All data generated or analysed during this study are included in this published article [and its supplementary information files].
