# Supplementary material for: Models of social prescribing to address non-medical needs in adults: a scoping review
Source: BMC Health Serv Res. 2023 Jun 15;23:642. doi: 10.1186/s12913-023-09650-x (PMC10268538; doi:10.1186/s12913-023-09650-x)
Supplement: Supplementary file 1 — Additional file 1. Search strategy. [file 12913_2023_9650_MOESM1_ESM.docx]

# Additional File I: Search strategy

Ovid MEDLINE(R) and Epub Ahead of Print, In-Process, In-Data-Review & Other Non-Indexed Citations, Daily and Versions(R) 1946 to June 29, 2021

Search conducted on 1 July 2021*.*

| **Search** | **Query** | **Records retrieved** |
| --- | --- | --- |
| #1 | ((social or community or community-based) adj3 (referral* or prescrib* or prescription*)).ti,ab,kf. | 2,957 |
| #2 | ((non-medical or non-clinical) adj2 (referral* or prescribing)).ti,ab,kf. | 162 |
| #3 | green prescrib*.ti,ab,kf. | 1 |
| #4 | (link worker or link workers).ti,ab,kf. | 84 |
| #5 | (System navigator* or care navigator* or community connector*).ti,ab,kf. | 89 |
| #6 | or/1-5 | 3239 |
