# Supplementary material for: Models of social prescribing to address non-medical needs in adults: a scoping review
Source: BMC Health Serv Res. 2023 Jun 15;23:642. doi: 10.1186/s12913-023-09650-x (PMC10268538; doi:10.1186/s12913-023-09650-x)
Supplement: Supplementary file 2 — Additional file 2. Data extraction instrument. [file 12913_2023_9650_MOESM2_ESM.docx]

**Additional file 2. Data extraction instrument**

| **Scoping Review Details** | |
| --- | --- |
| Scoping review title: | The use of social prescribing to address non-medical needs: a scoping review protocol |
| Review objectives: | To describe the types of social prescribing models used to connect people to non-medical services and supports |
| Review questions: | Primary question:  What types of social prescribing models are used to connect adults aged 18 years and older to non-medical services?  Secondary questions:  a) What are the contexts in which social prescribing programs have been delivered?  b) What population groups have been targeted/included?  c) What types of services/supports are referred to?  d) What staff are involved in social prescribing programs?  e) What funding mechanisms are used to support social prescribing programs?  f) What is the potential role of digital systems in social prescribing programs? |
| **Inclusion/Exclusion Criteria** | |
| Population | Adults aged 18 and over |
| Concept | Include:  Studies of programs with the primary focus of linking people with services and supports outside of the health system to meet their non-medical needs.  Exclude:  Studies that include an element of social prescribing as an adjunct to another intervention.  Studies that focus on medical system navigation without addressing non-medical needs. |
| Context | Include:  All health settings.  All non-health settings.  Exclude:  Studies of social prescribing in low- and middle-income countries as defined at www.oecd.org |
| Types of evidence source | Include:  Peer-reviewed full text literature reporting qualitative, quantitative, and mixed methods studies.  Grey literature including research reports, Masters and PhD theses (honours theses will be excluded), and unpublished clinical trials (where the research has not also been published in a peer-reviewed journal).  Conference abstracts with sufficient detail reported.  Exclude:  Opinion papers.  Research protocols.  Conference abstracts with insufficient detail reported. |
| **Evidence Source Details and Characteristics** | |
| Citation details (author/s, date, title) |  |
| Country |  |
| Context |  |
| Participant details (age/sex and number) |  |
| Type of research (quantitative, qualitative, mixed methods) |  |
| Type of source (peer-reviewed; grey literature; thesis) |  |
| **Details/Results extracted from source of evidence** (in relation to the concept of the scoping review) | |
| Terminology (social prescribing, community connecting, etc.) |  |
| Type of intervention: Target population(s) |  |
| Type of intervention: non-medical needs addressed in the intervention |  |
| Type of intervention: non-medical services prescribed |  |
| Type of intervention: Identification of available non-medical services (programme designers, link worker, etc.) |  |
| Type of intervention: referral process |  |
| Staff involved in the program and their role(s) |  |
| Funding mechanism |  |
| Digital system(s) used in the intervention |  |
