# Supplementary material for: Models of social prescribing to address non-medical needs in adults: a scoping review
Source: BMC Health Serv Res. 2023 Jun 15;23:642. doi: 10.1186/s12913-023-09650-x (PMC10268538; doi:10.1186/s12913-023-09650-x)
Supplement: Supplementary file 3 — Additional file 3. Data summary table. [file 12913_2023_9650_MOESM3_ESM.docx]

**Additional File 3: Data summary table**

| **Author**  **Date** | **Country** | **Context** | **Type of research** | **Type of source** | **Terminology** | **Target population** | **Non-medical needs** | **Non-medical services** | **Service identifying** | **Staff & role** | **Training** | **Funding** | **Digital systems** |
| --- | --- | --- | --- | --- | --- | --- | --- | --- | --- | --- | --- | --- | --- |
| Abram-sohn et al  2020 (108) | USA | Primary care | Mixed methods | Peer-reviewed | Community referral | People with cardio-vascular disease | Specific: Behaviour-al health | Specific: Behaviour-al Health | Program designers | Existing: HC staff (screen & refer) | Yes: SP process | None | Online database of services |
| Ackerman, Deyo & LoGerfo  2005 (109) | USA | Primary care | Quantitative | Peer-reviewed | Exercise referral | Older people | Specific:  Physical inactivity (Stage of readiness survey) | Specific: Physical activity | Program designers | Existing: HC staff (screen & refer) | Yes: SP process | None | None |
| Age UK  2011 (110) | UK | Primary care | Quantitative | Grey literature | Social prescribing | Older people | General | General | Program designers | Existing: HC staff (identification) & CBO staff (LW) | None | None | None |
| Aggar et al.  2021 (111) | Australia | Primary care & Community | Quantitative | Peer-reviewed | Social prescribing | People with mental health issues | General | General | Program designers | Existing: HC staff (identification)  New: LW role;  Support & follow-up | None | Govt. | None |
| Baines  2015 (51) | UK | Primary care | Mixed methods | Grey literature | Social prescribing | General | General | General | LW | Existing: HC staff (identification; referral form)  New: LW role & Volun-teer LW & Co-ordina-tor;  Support & follow-up | Yes: SP process | Govt. | Online referral platform |
| Baker & Irving  2016 (112) | UK | Primary care & Community | Mixed methods | Peer-reviewed | Social prescribing | People with dementia | Specific: isolation & loneliness | Specific: Arts services | Program designers | Existing: HC (identification) & CBO staff (LW) | None | Govt. | None |
| Bartlett-Prescott, Klesges & Kritchev-sky  2005 (52) | USA | Primary care & Community | Quantitative | Peer-reviewed | Health promotion referral | General | Specific: Behaviour-al health | Specific: Behaviour-al health | Program designers | Existing: HC (identification; referral form) & CBO staff (LW) | Yes: SP process | None | None |
| Bertotti et al.  2018 (39) | UK | Primary care | Mixed methods | Peer-reviewed | Social prescribing | At-risk population | General | General | LW | Existing: HC staff (identification)  New: LW role;  Support & follow-up | None  LW role: existing skills | Govt. | None |
| Bhatti et al.  2021 (53) | Canada | Primary care | Qualitative | Peer-reviewed | Social prescribing | General | General | General | HC staff & LWs | Existing: HC staff (identification)  New: LW role;  Support & follow-up | None | Govt. | None |
| Bickerton, Siegart & Marquez  2020 (113) | USA | Primary care | Quantitative | Peer-reviewed | Social needs screening  Community resource referral | At-risk population | General (screening tool) | General | Program designers | New: Student LW  Follow-up | None | None | EMRs (document referral) |
| Bird, Biddle & Powell  2019 (114) | UK | Primary care | Mixed methods | Peer-reviewed | Social prescribing | People with/at-risk of long term conditions | Specific: Physical inactivity | Specific: Physical activity | Program designers | Existing: HC staff (identification)  New: LW role | Yes: Health coaching | Govt. | EMRs (identify need) |
| Bleacher et al.  2020 (54) | USA | Primary care | Quantitative | Peer-reviewed | Community referral  Social needs screening | General | General (screening tool) | General | LW | Existing: HC staff (identification)  New: LW roles – HC and volun-teer | None | None | EMR (document screening results) |
| Blickem et al.  2013 (115) | UK | Community (online) | Qualitative | Peer-reviewed | Social prescribing  Community referral | People with long term conditions | General (screening tool) | General | Program designers | None: online  Self-referral | None | None | Online screening and referral platform |
| Boyle, Soraghan & Robinson  2017 (55) | UK | Tertiary care | None | Peer-reviewed | Social prescribing | General | General | General | Program designers | Existing: HC staff (screen & refer) | None | None | Online referral platform  Online database of services |
| Brandling et al.  2011 (56) | UK | Primary care | Mixed methods | Grey literature | Social prescribing | General | General | General | LW | Existing: HC staff (identification)  New: LW role;  Support & follow-up | None  LW role: existing skills | Govt. | None |
| Brown  2016 (57) | UK | Primary care | Mixed methods | Grey literature | Arts on prescription | General | General | Specific: Arts services | Program designers | Existing: HC staff (screen & refer)  Self-referral | None | None | None |
| Browne  1997 (116) | UK | Primary care | None | Peer-reviewed | Exercise by prescription | People who are sedentary | Specific: Physical inactivity | Specific: Physical activity | LW | Existing: HC staff, social services staff (referral)  New: LW role | None | None | None |
| Carnes et al.  2017 (40) | UK | Primary care | Mixed methods | Peer-reviewed | Social prescribing | At-risk population | General | General | LW | Existing: HC staff (identification)  New: LW role & Volun-teer LW;  Support & follow-up | Yes: SP process | Govt. | None |
| Carty et al.  2016 (117) | New Zealand | Multiple health & Community | Mixed methods | Peer-reviewed | Books on prescription | People with mental health issues | Specific:  Behaviour-al health | Specific: Self-help | Program designers | Existing: HC (identification) & Library staff LW | None | Govt. & Charity | Library system |
| Chng et al.  2021 (58) | UK | Primary care | Qualitative | Peer-reviewed | Social prescribing | General | General | General | LW | Existing: HC staff (identification)  New: LW role & Co-ordina-tor | None | Govt. | None |
| City & Hackney Clinical Commis-sioning Group  2015 (59) | UK | Primary care | Mixed methods | Grey literature | Social prescribing | General | General | General | Program designers | Existing: HC staff (identification)  New: LW role | Yes: Health Coaching | Govt. | None |
| Craig et al. 2008 (118) | UK | Tertiary care | Mixed methods | Peer-reviewed | Social outreach | At-risk population | General (screening tool) | General | Program designers | Existing: HC staff (identification)  New: LW role  Support & follow-up | None | Charity | EMR (document referral) |
| Crone, Johnston & Grant  2004 (60) | UK | Primary care | None | Peer-reviewed | Exercise referral | General | Specific:  Physical inactivity | Specific: Physical activity | Program designers | Existing: HC staff (identification)  New: LW role | None | Govt. | Online referral platform |
| Cusack et al.  2019 (119) | USA | Primary care & Community | Quantitative | Peer-reviewed | Screening/ referral for SDH | Veterans | Specific: Homeless-ness (screening tool) | Specific: Homeless services | Program designers | Existing: HC (identification) & CBO staff (LW) | None | Govt | EMR (embedded screening tool) |
| Dayson & Bashir  2014 (121) | UK | Primary care | Mixed methods | Grey literature | Social prescribing | People with long term conditions | General | General | Program designers | Existing: HC staff (identification)  New: LW role | None | Govt. | None |
| Dayson & Bennett  2016 (124) | UK | Primary care & Community | Mixed methods | Grey literature | Social prescribing | General | General | General | LW | Existing: HC staff (identification)  New: LW role & Volun-teer LW;  Support & follow-up | None | Govt. | None |
| Dayson  2017 (120) | UK | Primary care & Community | Mixed methods | Peer-reviewed | Social prescribing | People with long term conditions | General | General | Program designers | Existing: HC staff (identification)  New: LW role | None | Govt. | None |
| Dayson & Leather  2018 (62) | UK | Primary care | Mixed methods | Grey literature | Social prescribing | General | General | General | LW | Existing: HC staff (identification)  New: LW role;  Support & follow-up | None | Govt. | None |
| Dayson et al.  2016 (122) | UK | Primary care | Mixed methods | Grey literature | Social prescribing | People with long term conditions | General | General | Program designers | Existing: HC staff (identification)  New: LW role | None | Govt. | None |
| Dayson, Bashir & Pearson  2013 (123) | UK | Primary care | Quantitative | Grey literature | Social prescribing | People with long term conditions | General | General | Program designers | Existing: HC staff (identification)  New: LW role | None | Govt. | None |
| Dayson, Fraser & Lowe  2020 (61) | Two prog-rams  UK | Primary care & Community  Primary care & Community | Qualitative | Peer-reviewed | Social prescribing  Social prescribing | People with long term conditions  People with long term conditions | General  General | General  General | Not stated  Not stated | Existing: HC staff (identification)  New: LW role; Support& follow up  Existing: HC staff (identification) New: LW role; Support& follow up | None  None | Social impact bond  Govt. | None  None |
| Dayson, Painter & Bennett  2020 (125) | UK | Secondary care | Qualitative | Peer-reviewed | Social prescribing | People with mental health issues | General | General | Program designers | Existing: HC staff (identification) & HC staff LW;  Support & follow-up | None | Govt. | None |
| Din et al.  2015 (126) | UK | Primary care | Qualitative | Peer-reviewed | Exercise referral | People who are sedentary | Specific: Physical inactivity | Specific: Physical activity | Program designers | Existing: HC staff (identification)  New: LW role;  Support & follow-up | None | Govt. | None |
| Dinan et al.  2006 (127) | UK | Primary care | Quantitative | Peer-reviewed | None | Older people | Specific: Frailty | Specific: Physical activity | Program designers | Existing: HC staff (screen & refer) | None | Govt. | None |
| Duffin  2016 (128) | UK | Multiple health & Community | None | Peer-reviewed | Social prescribing | People who have cancer | General | General | LW | Existing: HC staff (identification; referral form)  New: LW role;  Support & follow-up  Self-referral | None  LW role: existing skills | None | Case manage-ment system |
| Edwards, Souter & Best  2018 (129) | UK | Secondary care & Community | Mixed methods | Peer-reviewed | Community connecting | People in recovery from drug & alcohol problems | General | General | Program designers | Existing: HC staff (identification)  New: LW role | Yes: SP process | Govt. | None |
| Elston et al.  2019 (130) | UK | Multiple health & Community | Quantitative | Peer-reviewed | Social prescribing | Older people | General | General | LW | Existing: HC & CBO staff (identification)  New: LW role;  Support & follow-up | Yes: Health coaching | Govt. | None |
| Faulkner  2004 (63) | UK | Primary care | Qualitative | Peer-reviewed | None | General | General | General | LW | Existing: HC staff (identification)  New:  Volun-teer LWs  Support | Yes: Health coaching | None | None |
| Ferguson  2018 (64) | UK | Primary care | Mixed methods | Grey literature | Social prescribing | General | General (screening tool) | General | LW | Existing: HC staff (identification)  New: LW role;  Support & follow-up  Self-referral | Yes: Health coaching | Govt. | Case manage-ment system |
| Fiori et al.  2019 (65) | USA | Primary care | Quantitative | Peer-reviewed | Social needs screening/ referral | General | General (screening tool) | General | LW | Existing: HC staff (identification)  New: HC staff LW & Coordi-nator | Yes: SP process | Govt. | EMR (embed screening tool) |
| Fixsen et al.  2020 (9) | UK | Primary care | Qualitative | Peer-reviewed | Social prescribing | People with cardio-vascular disease | General | General | Program designers | Existing: HC staff (identification)  New: LW role;  Support & follow-up | Yes: Health coaching | Govt. | EMR (access) |
| Fixsen, Barrett & Shimo-novich  2021 (66) | Three pro-grams  UK | Primary care & Community  Primary care & Community  Primary care & Community | Qualitative | Peer-reviewed | Social prescribing  Social prescribing  Social prescribing | General  General  Older people | General  General  General | General  General  General | LW  LW  LW | Existing: HC staff (identification) New: LW role  Self-referral  Existing: HC staff (identification) New: LW role  Existing: HC staff (identification) New: LW role | None  LW role: existing skills  None LW role: existing skills  None LW role: existing skills | Govt.  Govt.  Govt | Telehealth  Telehealth  Telehealth |
| Flannery et al.  2014 (131) | UK | Primary care | Mixed methods | Grey literature | Exercise on prescription | People who are sedentary | Specific: Physical inactivity | Specific: Physical activity | Program designers | Existing: HC staff (screen & refer) | None | None | None |
| Fleming, Bryce et al.  2020 (132) | UK | Primary care | Mixed methods | Peer-reviewed | Social prescribing | People who are sedentary | Specific: Physical inactivity | Specific: Physical activity | Program designers | Existing: HC staff (screen & refer)  Self-referral | None | None | None |
| Fleming, Wellington et al.  2020 (133) | UK | Primary care | Mixed methods | Peer-reviewed | Social prescribing | People who are sedentary | Specific: Physical inactivity | Specific: Physical activity | Program designers | Existing: HC staff (screen & refer)  Self-referral | None | None | None |
| Flocke, Gordon & Pomiecko  2006 (67) | USA | Primary care | Mixed methods | Peer-reviewed | Community health promotion | General | Specific: Behavioural health | Specific: Behavioural health | Program designers | Existing: HC staff (screen & refer; referral form) | None | None | Online database of services |
| Foster et al.  2021 (134) | UK | Community | Mixed methods | Peer-reviewed | Social prescribing | People who experience or are at risk of loneliness | Specific:  Loneliness | General | LW | Existing: HC staff (identification)  New: LW role & Volun-teer LW;  Support & follow-up  Self-referral | None | Charity | None |
| Friedli, Themessl-Huber & Butchart  2012 (68) | UK | Primary care | Mixed methods | Grey literature | Social prescribing | General | General | General | LW | Existing: HC staff (identification)  New: LW role;  Support & follow-up | None | None | None |
| Fuller  2017 (69) | UK | Primary care | Quantitative | Peer-reviewed | Social prescribing | General | General | General | LW | Existing: HC staff (identification)  New: LW role | None | Govt. | None |
| Galaviz, Levesque & Kotecha  2012 (135) | Canada | Primary care | Quantitative | Peer-reviewed | Physical activity referral | People who are sedentary | Specific: Physical inactivity | Specific: Physical activity | Program designers | Existing: HC staff (screen & refer) | Yes: Health coaching | None | None |
| Garg et al.  2015 (136) | USA | Primary care | Quantitative | Peer-reviewed | Community service referral  Social needs screening | Mothers | General | General | Program designers | Existing: HC staff (screen & refer) | None | None | None |
| Gaskin et al.  2017 (137) | Australia | Tertiary care | Quantitative | Peer-reviewed | None | People who have cancer | Specific:  Physical inactivity | Specific: Physical activity | Program designers | Existing: HC staff (screen & refer) | Yes: SP process | None | None |
| Gibson, Pollard & Moffatt  2021 (138) | UK | Primary care | Qualitative | Peer-reviewed | Social prescribing | People with long term conditions | General | General | LW | Existing: HC staff (identification)  New: LW role;  Support & follow-up | None | None | None |
| Giebel et al.  2020 (139)  (updated to 2022) | UK | Multiple health & Community | Qualitative | Peer-reviewed | Community connecting | People with mental health issues | General | General | LW | Existing: HC staff (identification)  New: LW role & Volun-teer LW;  Support & follow-up  Self-referral | None | Govt. | None |
| Giebel et al.  2021 (140) | UK | Primary care | Quantitative | Peer-reviewed | Social prescribing | People with dementia/ carer | General | General | Program designers | Existing: HC staff (identification)  New: LW role;  Support  Self-referral | None | Govt. | None |
| Gold et al.  2017 (70) | USA | Primary care | Qualitative | Peer-reviewed | Community resource referral | General | General (screening tool) | General | Program designers | Existing: HC staff (screen & refer) | None | None | EMR (document screening results) |
| Golubinski et al.  2020 (71) | Germa-ny | Primary care & Secondary care | Quantitative | Peer-reviewed | Social prescribing | General | General | General | LW | Existing: HC staff (identification; referral form)  New: HC staff LW  Self-referral | None | Private company | None |
| Grant et al.  2000 (72) | UK | Primary care | Quantitative | Peer-reviewed | None | General | General | General | LW | Existing: HC staff  New: LW role;  Support & follow-up | Yes: SP process | Govt. | None |
| Grayer et al.  2008 (73) | UK | Primary care | Quantitative | Peer-reviewed | Social prescribing | General | General | General | LW | Existing: HC staff (identification)  New: LW role;  Support & follow-up | None | None | None |
| Hager et al.  2020 (41) | USA | Primary care & Tertiary care | None | Peer-reviewed | Food resource referral | At risk population | Specific: Food insecurity (screening tool) | Specific: Food bank | Program designers | Existing: HC staff (identification; referral form) & CBO staff (LW) | Yes: SP process | None | EMR (embedded screening tool) |
| Halliday & Wilkinson  2009 (42) | UK | Multiple health & Community | Mixed methods | Peer-reviewed | None | At-risk population | General | General | LW | Existing: HC staff (identification)  New: LW role;  Support & follow-up | Yes: Health coaching | Govt. | None |
| Hamilton-West et al.  2019 (74) | Two pro-grams  UK | Primary care  Primary care | Mixed methods | Peer-reviewed | Social prescribing  Social prescribing | People with mental health issues  General | General  General | General  General | LW  Program designers | Existing: HC staff (identification; referral form) New: LW role & Coordinator; Support & follow-up  Self-referral  Existing: HC staff (identification) New: LW role & Coordinator; Support & follow-up  Self-referral | Yes: SP process  Yes: SP process | Govt.  Govt. | None  Online database of services |
| Hanlon et al.  2021 (75) | UK | Primary care | Qualitative | Peer-reviewed | Social prescribing | General | General | General | LW | Existing: HC staff (identification)  New: LW role;  Support & follow-up  Self-referral | Yes: SP process | Govt. | None |
| Harrison, Roberts & Elton  2005 (141) | UK | Primary care | Mixed methods | Peer-reviewed | Exercise referral | People who are sedentary | Specific: Physical inactivity | Specific:  Physical activity | Program designers | Existing: HC staff (identification; referral form)  New: LW role | None  LW role: existing skills | Govt. | None |
| Hassan et al.  2015 (142) | USA | Community (online) | Quantitative | Peer-reviewed | Community resource referral | Young people | General (screening tool) | General | Program designers | None (online)  Self-referral | None | None | Online screening & referral platform |
| Hazeldine et al.  2021 (76) | Two prog-rams  UK | Primary care & Community  Primary care & Community | Qualitative | Peer-reviewed | Social prescribing  Social prescribing | General  General | General  General | General  General | LW  LW | Existing: HC staff (identification) New: LW role  Existing: HC staff (identification) New: LW role;  Support & follow-up | None  None | Govt.  CBO | None  None |
| Heijnders & Meijs  2018 (77) | Nether-lands | Primary care & Community | Qualitative | Peer-reviewed | Social prescribing | General | General | General | LW | Existing: HC staff (identification)  New: LW role;  Support & follow-up | None | None | Referral document-ted in GP information system |
| Holding et al.  2020 (143) | UK | Community | Qualitative | Peer-reviewed | Social prescribing | People who experience or are at risk of loneliness | Specific: Loneliness | General | LW | Existing: HC staff (identification)  New: LW role & Volun-teer LW;  Support & follow-up  Self-referral | Yes: SP process | Charity | None |
| Horner  2019 (144) | UK | Community | None | Peer-reviewed | Social prescribing | Young people | General | General | LW | Existing: HC staff (identification)  New: LW role;  Support & follow-up | None | Govt. | None |
| Isaacs et al.  2007 (145) | UK | Primary care | Quantitative | Peer-reviewed | Exercise referral | People who are sedentary | Specific: Physical inactivity | Specific: Physical activity | Program designers | Existing: HC staff (screen & refer) | None | None | None |
| Ismail et al.  2020 (146) | UK | Multiple health & Community | Quantitative | Peer-reviewed | None | People with long term conditions | General | General | LW | Existing: HC staff (identification; referral form)  New: HC staff LW;  Support & follow-up | None | None | Online referral form |
| James et al.  2009 (147) | UK | Primary care | Quantitative | Peer-reviewed | Physical activity referral | People who are sedentary | Specific: Physical inactivity | Specific: Physical activity | Program designers | Existing: HC staff (identification)  New: LW role;  Support & follow-up | None | Govt. | None |
| Jensen & Grounds  2016 (78) | USA | Para-medicine | None | Peer-reviewed | Social needs referral | General | General | General | LW | Existing: HC staff (identification) & CBO staff (LW);  Support & follow-up | Yes: SP process | None | Case manage-ment system |
| Kellezi et al.  2019 (79) | UK | Primary care | Mixed methods | Peer-reviewed | Social prescribing | General | General | General | LW | Existing: HC staff (identification)  New: LW role;  Support & follow-up | None | Govt. | None |
| Kiely et al.  2021 (148) | UK | Primary care | Mixed methods | Peer-reviewed | Social prescribing | People with long term conditions/ multi-morbidity | General | General | LW | Existing: HC staff (identification)  New: LW role; Support & follow-up | None | None | None |
| Kimberlee  2016 (80) | UK | Primary care | Mixed methods | Grey literature | Social prescribing | General | General | General | LW | Existing: HC staff (identification)  New: LW role;  Support & follow-up | None | Govt. | None |
| Kimberlee et al.  2014 (149) | UK | Primary care | Mixed methods | Grey literature | Social prescribing | People with mental health issues | General | General | LW | Existing: HC staff (identification)  New: LW role;  Support & follow-up | None  LW role: existing skills | Charity | None |
| Kulie et al.  2021 (81) | USA | Tertiary care | Quantitative | Peer-reviewed | Social needs referral | General | General (screening tool) | General | Program designers | New: LW role | None | None | Online screening & referral platform |
| Lachance, Malinovich & Garrity  2016 (150) | Three prog-rams  USA | Primary care & Community  Primary care & Community  Primary care & Community | Qualitative | Peer-reviewed | None  None  None | Mothers  People with mental health issues  People with long term conditions | Healthy eating  General  General | Food prescrip-tion  General  General | Program designers  Program designers  Program designers | Staffing unclear  Staffing unclear  Staffing unclear | None  None  None | Charity  Charity  Charity | None  None  None |
| Lindau et al.  2016 (47) | USA | Primary care & Tertiary care | Quantitative | Peer-reviewed | Community resource referral | General | General | General | Program designers | Existing: HC staff (refer) | None | None | IT Platform interfaced with Electronic Health Records to generate prescription |
| Lindau et al.  2019 (48) | USA | Primary care & Tertiary care | Quantitative | Peer-reviewed | Community resource referral | General | General | General | Program designers | Existing: HC staff (screen & refer) | None | None | IT Platform interfaced with Electronic Health Records to generate prescription |
| Liu et al.  2021 (151) | USA | Primary care & Tertiary care | None | Peer-reviewed | Social needs referral | At risk population | General | General | Program designers | Existing: HC staff (identification)  New: HC staff LW;  Support & follow-up | Yes: SP process | None | Online referral platform |
| Loftus, McCauley & McCarron  2017 (152) | UK | Primary care | Quantitative | Peer-reviewed | Social prescribing | Older people | General | General | Program designers | Existing: HC staff (identification)  New: LW role; Support & follow-up | None | None | None |
| Longwill  2014 (82) | UK | Primary care | Mixed methods | Grey literature | None | General | General | General | CBO staff LW | Existing: HC (identification) & CBO staff (LW);  Support & follow-up | None  LW role: existing skills | Govt. | Non-medical referral integrated into existing referral system |
| Losonczy et al.  2017 (83) | USA | Tertiary care | Quantitative | Peer-reviewed | None | General | General | General | Program designers | New: Volun-teers LW role | Yes: SP process | None | None |
| Lowrie et al.  2021 (153) | UK | Tertiary care | Quantitative | Peer-reviewed | Social prescribing  Non-medical prescribing | At-risk population | General | General | LW | Existing: HC staff (identification)  New: LW role; Support & follow-up | None | Govt. | None |
| Makelarski et al.  2020 (49) | USA | Primary care | Mixed methods | Peer-reviewed | Community resource referral | People at risk of cardiovascular disease | General | General | Program designers | Existing: HC staff (refer) | None | None | IT Platform interfaced with Electronic Health Records to generate prescription |
| Marco et al.  2018 (154) | Australia | Tertiary care | Mixed methods | Peer-reviewed | None | People with cancer | Specific:  Cancer Infor-mation & support | Specific:  Cancer Council support services | Program designers | Existing: HC staff (screen & refer; referral form) | Yes: SP process | None | None |
| Marpadga et al.  2019 (50) | USA | Tertiary care | Qualitative | Peer-reviewed | Social needs screening/ referral | People with long term health conditions | Specific:  Food insecurity (screening tool) | Specific:  Commu-nity-based food resources | Program designers | New: Volun-teer LW role;  Support | Yes: SP process | None | None |
| Martin & Woolf-May  1999 (155) | UK | Primary care | Mixed methods | Peer-reviewed | Exercise prescription | People who are sedentary | Specific: Physical inactivity | Specific: Physical activity | Program designers | Existing: HC staff (identification)  New: LW role;  Support & follow-up | None | None | None |
| Maughan et al.  2016 (156) | UK | Primary care & Community | Quantitative | Peer-reviewed | Social prescribing | People with mental health issues | General | General | Program designers | Existing: HC staff (identification)  New: LW role; Support & follow-up | Yes: SP process | Charity | None |
| McMahon  2013 (84) | UK | Primary care | Quantitative | Grey literature | Social prescribing | General | General | General | Program designers | Existing: HC staff (identification)  New: LW role;  Support & follow-up  Self-referral | None | None | None |
| Mekni & Haynes  2020 (85) | USA | Community (online) | None | Peer-reviewed | Community resource referral | General | General (screening tool) | General | Program designers | None (online)  Self-referral | None | None | Online screening & referral platform |
| Mercer et al.  2017 (86) | UK | Primary care | Mixed methods | Grey literature | Social prescribing | General | Genera | General | LW | Existing: HC staff (identification)  New: LW role;  Support & follow-up | None | Govt. | None |
| Mercer et al.  2019 (157) | UK | Primary care | Quantitative | Peer-reviewed | Social prescribing | At-risk population | General | General | LW | Existing: HC staff (identification)  New: LW role; Support & follow-up | None | Govt. | None |
| Mills  2008 (158) | UK | Primary care | Mixed methods | PhD Thesis | Exercise referral | People who are sedentary | Specific: Physical inactivity | Specific:  Physical activity | Program designers | Existing: HC staff (identification)  New: LW role;  Support & follow-up | None | Govt. | None |
| Mills et al.  2013 (159) | UK | Primary care | Mixed methods | Peer-reviewed | Exercise referral | People who are sedentary | Specific: Physical inactivity | Specific:  Physical activity | Program designers | Existing: HC staff (identification)  New: LW role;  Support & follow-up | None | None | None |
| Moffatt et al.  2017 (160) | UK | Primary care & Community | Qualitative | Peer-reviewed | Social prescribing | Older people | General | General | LW | Existing: HC staff (identification)  New: LW role; Support & follow-up | Yes: Health coaching | Social Impact Bond | None |
| Moore et al.  2013 (161) | UK | Primary care & Community | Mixed methods | Peer-reviewed | Exercise referral | People who are sedentary | Specific: Physical inactivity | Specific: Physical activity | Program designers | Existing: HC staff (identification)  New: LW role; Support & follow-up | Yes: Health coaching | Govt. | None |
| Morton, Ferguson & Baty  2015 (162) | UK | Secondary care & Community | Quantitative | Peer-reviewed | Social prescribing | People with mental health issues | General | General | Program designers | Existing: HC staff (screen & refer)  Self-referral | None | Govt. | None |
| Mulligan et al.  2020 (87) | Canada | Primary care & Community | None | Peer-reviewed | Social prescribing | General | General | General | Program designers | Existing: HC staff (identification) & CBO staff (LW) | None | None | None |
| Nehme et al.  2021 (88) | Three prog-rams  USA | Primary care  Primary care  Primary care | Qualitative | Peer-reviewed | Social needs screening & referral  Social needs screening & referral  Social needs screening & referral | General  General  General | General  General  General | General  General  General | LW  LW  LW | Existing: HC staff (identification) & HC staff LW role  Existing: HC staff (identification) & HC staff LW role  Existing: HC staff (identification) & HC staff LW role | None  None  None | None  None  None | None  None  Screening & referral platform |
| Nguyen et al.  2016 (163) | USA | Primary care | Quantitative | Peer-reviewed | None | At-risk population | General (screening tool) | General | LW | New: Volun-teer LW | Yes: SP process | None | Online database of services |
| Page-Reeves et al.  2016 (89) | USA | Tertiary care | Quantitative | Peer-reviewed | Screening/ referral for SDH | General | General | General | Program designers | Existing: HC staff (screen & refer) | Yes: SP process | None | None |
| Palmer et al.  2017 (164) | UK | Primary care & Community | Mixed methods | Grey literature | Social prescribing | People with long term conditions | General (screening tool) | General | Program designers | Existing: HC staff (identification)  New: LW role;  Support & follow-up  Self-referral | None | Govt. | Online referral platform |
| Payne, Walton & Burton  2020 (90) | USA | Primary care & Community | Qualitative | Peer-reviewed | Social prescribing | General | General | General | LW | Existing staff: HC staff (identification)  New: LW role; Support & follow-up | None | Charity | None |
| Pescheny et al.  2019 (165) | UK | Primary care | Quantitative | Peer-reviewed | Social prescribing | People with long term conditions | General | General | LW | Existing: HC staff (identification)  New: LW role; Support & follow-up | None | Govt. | None |
| Pescheny, Pappas & Randhawa  2018 (92) | UK | Primary care | Qualitative | Peer-reviewed | Social prescribing | General | General | General | LW | Existing: HC staff (identification)  New: LW role; Support & follow-up | None | Govt. | None |
| Pescheny, Randhawa & Pappas  2018 (91) | UK | Primary care | Qualitative | Peer-reviewed | Social prescribing | General | General | General | LW | Existing: HC staff (identification)  New: LW role; Support & follow-up | Yes: Health coaching | Govt. | None |
| Power-Hays et al.  2020 (166) | USA | Tertiary care | Quantitative | Peer-reviewed | Screening/ referral for SDH | At risk population | General (screening tool) | General | Program designers | Existing: HC staff (screen & refer)  New: Coordinator  Follow-up | None | None | Online referral database |
| Pratt et al.  2015 (167) | UK | Primary care | Mixed methods | Peer-reviewed | None | People with long term conditions / multi-morbidity | General (screening tool) | General | Program designers | Existing: HC staff (screen & refer) | Yes: SP process | Govt. | Online screening tool |
| Prior  2019 (168) | UK | Multiple health & Community | Mixed methods | PhD Thesis | Exercise referral | People who are sedentary | Specific: Physical inactivity | Specific: Physical activity | Program designers | Existing: HC staff (identification)  New: LW role;  Support & follow-up | None  LW role: existing skills | Govt. | None |
| Prior et al.  2019 (169) | UK | Multiple health & Community | Quantitative | Peer-reviewed | Exercise referral | People who are sedentary | Specific: Physical inactivity | Specific: Physical activity | LW | Existing staff: HC staff (identification)  New: LW role; Support & follow-up | None | None | None |
| Pruitt et al.  2018 (170) | USA | Multiple health & Community | Quantitative | Peer-reviewed | Social service referral | At-risk population | General | General | Program designers | Existing: HC staff & Mana-ged care staff (LW); Follow-up | None | Mana-ged Care Organi-sation | Online database of services |
| Randall  2015 (171) | UK | Multiple health & Community | None | Peer-reviewed | Social prescribing | At-risk population | General | General | Program designers | Existing: HC staff (identification)  New: LW role  Self-referral | None | None | None |
| Redmond et al.  2019 (172) | UK | Primary care | Qualitative | Peer-reviewed | Social prescribing | People with mental health issues | General | Specific: Commu-nity art program | Program designers | Existing: HC staff (screen & refer) | None | None | None |
| Rhodes & Bell  2021 (93) | UK | Primary care & Community | Qualitative | Peer-reviewed | Social prescribing | General | General | General | LW | Existing: HC staff (identification)  New: LW role; Support & follow-up  Self-referral | Yes: SP process | Govt. | None |
| Salvatore et al.  2020 (94) | USA | Secondary care & Community | None | Peer reviewed | Social needs screening | General | General (screening tool) | General | Program designers | Existing: Social services staff LW (screen & refer);  Support & follow-up | None | Charity | Online screening & referral platform |
| Sand  2021 (173) | USA | Primary care | Quantitative | Peer-reviewed | Social needs screening & referral | At-risk population | General (screening tool) | General | Program designers | Existing: HC staff (screen & refer)  New: Volun-teer LW | Yes: SP process | None | Online screening tool |
| Sanderson et al.  2021 (174) | USA | Primary care | Quantitative | Peer-reviewed | Social needs screening & referral | At-risk population | General (screening tool) | General | Program designers | Existing: HC staff (refer)  New: HC staff LW | None | Govt. | Online screening & referral platform |
| Sharan et al.  2017 (175) | USA | Community | None | Peer-reviewed | Social service referral | At-risk population | General | General | Program designers | Existing: CBO staff LW | Yes: SP process | None | Online referral platform |
| Simpson et al.  2020 (176) | UK | Secondary care & Community | Qualitative | Peer-reviewed | Social prescribing | People with long term conditions | General (screening tool) | General | LW | Existing: HC staff (identification)  New: LW role; Support & follow-up | None | None | None |
| Skivington et al.  2018 (95) | UK | Primary care | Qualitative | Peer-reviewed | Social prescribing | General | General | General | LW | Existing: HC staff (identification)  New: LW role; Support & follow-up | None | Govt. | None |
| Soraghan et al.  2016 (177) | UK | Tertiary care | Quantitative | Peer-reviewed | Social prescribing | Older people | General | General | Program designers | Existing: HC staff (screen & refer) | None | None | Database of existing services |
| Spain et al.  2021 (178) | USA | Primary care | Qualitative | Peer-reviewed | Social needs screening & referral | Parents | General (screening tool) | General | Program designers | Existing: HC staff (screen & refer) | None | None | Online screening tool |
| Stenmark et al.  2018 (179) | USA | Primary care | Quantitative | Peer-reviewed | Social needs referral | At-risk population | Specific: Food insecurity (screening tool) | Specific: Commu-nity-based food resources | Program designers | Existing: HC staff (identification) & CBO staff (LW; referral form) | Yes: SP Process | Charity | Online screening & referral platform |
| Stevens et al.  2015 (180) | USA | Tertiary care | Quantitative | Peer-reviewed | Social service referral | At-risk population | General (screening tool) | General | Program designers | Existing: HC staff (identification)  New: LW role; Support & follow-up | Yes: Health coaching | None | Online screening platform |
| Stickly & Hui  2012a (181) | UK | Multiple health & Community | Qualitative | Peer-reviewed | Social prescribing  Arts on prescription | People with mental health issues | Specific:  Social isolation | Specific: Commu-nity-based arts program | Program designers | Existing: HC staff (screen & refer) | None | Govt. & Charity | None |
| Stickly & Hui  2012b (182) | UK | Multiple health & Community | Qualitative | Peer-reviewed | Social prescribing  Arts on prescription | People with mental health issues | Specific:  Social isolation | Specific: Commu-nity-based arts program | Program designers | Existing: HC staff (screen & refer) | None | Govt. & Charity | None |
| Stirrat, McCallion & Youell  2013 (96) | UK | Primary care | Mixed methods | Grey literature | Green prescribing | General | Specific: Physical inactivity | Specific:  Physical activity | Program designers | Existing: HC staff (screen & refer) | None | None | None |
| Swift  2017 (97) | UK | Primary care & Community | Quantitative | Peer-reviewed | Social prescribing | General | General | General | LW | Existing: HC staff (identification)  New: LW role; Support & follow-up  Self-referral | Yes: Health coaching | Govt. | EMR (document referral) |
| Tung et al.  2020 (98) | USA | Primary care & Tertiary care | Quantitative | Peer-reviewed | Community resource referral | General | General | General | Program designers | Existing: HC staff (refer) | None | None | IT Platform interfaced with Electronic Health Records to generate prescription |
| Vest et al.  2019 (99) | USA | Primary care & Tertiary care | Quantitative | Peer-reviewed | Wraparound service referral | General | General | Specific: Wraparound services | Program designers | Existing: HC staff (refer) | None | None | Machine learning to generate risk stratification report |
| Vogelpoel & Jarrold  2014 (183) | UK | Primary care | Mixed methods | Peer-reviewed | Social prescribing | Older people | Specific: Social isolation | Specific: 12-week program-me | Program designers | Existing: HC staff (identification)  New: LW role; Follow-up | Yes: SP process | Charity | None |
| Wakefield et al.  2020 (184) | UK | Primary care | Qualitative | Peer-reviewed | Social prescribing | People with long term conditions | General | General | LW | Existing: HC staff (identification)  New: LW role; Follow-up  Self-referral | None | Govt. | None |
| Wallace et al.  2020 (100) | USA | Tertiary care | Mixed methods | Peer-reviewed | Social needs screening | General | General (screening tool) | General | Program designers | Existing: HC staff (identification) & CBO staff LW;  Follow-up | None | None | Online screening & referral platform |
| Ward et al.  2020 (101) | UK | Primary care | Mixed methods | Peer-reviewed | Social prescribing | General | General | General | Program designers | Existing: HC staff (identification)  New: Student LW role; Follow-up | Yes: SP process | None | None |
| White, Cornish & Kerr  2017 (102) | Three prog-rams  UK | Primary care  Primary care  Primary care | Qualitative | Peer-reviewed | Social prescribing  Social prescribing  Social prescribing | General  Carers  People with mental health issues | Behavioural health  General  Behavioural health | Behavioural health  General  Behavioural health | LW  LW  LW | Existing: HC staff (identification) & CBO staff LW role; Support & follow-up  Existing: HC staff (identification) & CBO staff LW role; Support & follow-up  Existing: HC staff (identification) & CBO staff LW role; Support & follow-up | None  None  None | None  None  None | None  None  None |
| Whitelaw et al.  2017 (103) | UK | Primary care | Qualitative | Peer-reviewed | Social prescribing | General | General | General | Program designers | Existing: HC staff (identification)  New: LW role;  Support & follow-up  Self-referral | None | Govt. | Online referral platform |
| Wildman, Moffatt, Penn et al.  2019 (185) | UK | Primary care | Qualitative | Peer-reviewed | Social prescribing | People with long term conditions | General | General | LW | Existing: HC staff (identification)  New: LW role;  Support & follow-up | Yes: Health coaching | Govt. | None |
| Wildman, Moffatt, Steer et al.  2019 (186) | UK | Primary care | Qualitative | Peer-reviewed | Social prescribing | People with long term conditions | General | General | LW | Existing: HC staff (identification)  New: LW role;  Support & follow-up | Yes: Health coaching | Govt. | None |
| Wilkinson et al.  2021 (187) | UK | Primary care | None | Peer-reviewed | Social prescribing | Older people | General | General | Program designers | Existing: HC staff (identification)  New: LW role & Volun-teer LW;  Support & follow-up | Yes: SP process | Charity | None |
| Wilson et al.  2010 (188) | USA | Primary care | Quantitative | Peer-reviewed | Prescription for health | People with unhealthy lifestyles | Specific: Behaviou-ral health | Specific: Behaviou-ral health | Program designers | Existing: HC staff (screen & refer) | None | None | EMR (prompt & referral) |
| Wood et al.  2021 (104) | UK | Primary care & Community | Qualitative | Peer-reviewed | Social prescribing | General | General | General | LW | Existing: HC staff (identification)  New: LW role;  Support & follow-up  Self-referral | None | CBO | None |
| Woodall et al.  2018 (105) | UK | Primary care | Mixed methods | Peer-reviewed | Social prescribing | General | General | General | LW | Existing: HC staff (identification)  New: LW role;  Support & follow-up  Self-referral | None | None | None |
| Wu et al.  2019 (189) | USA | Primary care & Community | Quantitative | Peer-reviewed | Screening/ referral for SDH | At risk population | General | General | Program designers | Existing: HC (identification) & CBO staff (LW);  Support & follow-up | None | None | Online referral platform |
| Yalçin, Moreno & DiPaola  2020 (106) | Canada | Community (Online) | None | Peer-reviewed | Social prescribing | General | General | Specific: Art prescription | Program designers | None | None | None | Artificial intelli-gence |
| Zhu, Ahluwalia & Laws  2020 (107) | USA | Primary care & Tertiary care | Qualitative | Peer-reviewed | Social referral | General | General | General | Program designers | Existing: HC staff (identification)  New: Volun-teer LW;  Support & follow-up | None | None | None |

Acronyms: SDH=Social Determinants of Health; HC=Healthcare Staff; CBO=Community-Based Organisation staff; LW=Link Worker; Govt=Government; EMR=Electronic Medical Record
